# Supplementary material for: Are objective measures of physical capability related to accelerated epigenetic age? Findings from a British birth cohort
Source: BMJ Open. 2017 Nov 1;7(10):e016708. doi: 10.1136/bmjopen-2017-016708 (PMC5695310; doi:10.1136/bmjopen-2017-016708)
Supplement: Supplementary file 1 [file bmjopen-2017-016708supp001.pdf]

Table S1: Association of age acceleration with physical capability at age 53 for NSHD participants

		Blood (n=152)			Buccal (n=790)		
**Variable**	Model[1]	Regression Coefficient (difference per year AA)	95% confidence interval	p-value	Regression Coefficient (difference per year AA)	95% confidence interval	p-value
**Grip strength (kg)**	Unadjusted	0.05	-0.25,0.36	0.73	-0.015	-0.12,0.09	0.78
	Adjusted for age, height, BMI	0.07	-0.21,0.36	0.61	-0.004	-0.10,0.10	0.94
	Adjusted for height, BMI, smoking, education and SEP	0.19	-0.14,0.51	0.26	0.010	-0.09,0.11	0.85
**Chair rise speed (stands/minute)**	Unadjusted	-0.29	-0.65,0.07	0.11	0.0001	-0.12,0.12	0.99
	Adjusted for age, height, BMI	-0.25	-0.60,0.09	0.15	0.025	-0.09,0.14	0.68
	Adjusted for height, BMI, smoking, education and SEP	-0.19	-0.57,0.18	0.31	0.002	-0.12,0.12	0.97
**Balance time, eyes closed (log seconds)**	Unadjusted	0.003	-0.02,0.03	0.81	0.003	-0.01,0.01	0.49
	Adjusted for age, height, BMI	0.005	-0.02,0.03	0.69	0.005	-0.003,0.013	0.20
	Adjusted for height, BMI, smoking, education and SEP	0.004	-0.02,0.03	0.71	0.004	-0.003,0.012	0.27
**Composite score**	Unadjusted	0.002	-0.01,0.01	0.77	0.001	-0.003,0.006	0.60
	Adjusted for age, height, BMI	0.003	-0.01,0.02	0.63	0.003	-0.002,0.007	0.22
	Adjusted for height, BMI, smoking, education and SEP	0.01	-0.01,0.02	0.39	0.002	-0.003,0.007	0.38

[1] For each of the four physical capability outcome measures, we ran three models, first unadjusted, then adjusted for height and BMI, then adjusted for height, BMI, smoking, education and both adult and childhood SEP

Table S2: Association of age acceleration at 53 with physical capability at age 60-64 for NSHD participants

		**Blood (n=152)**			**Buccal (n=790)**		
**Variable**	Model[2]	Regression Coefficient (difference per year AA)	95% confidence interval	p-value	Regression Coefficient (difference per year AA)	95% confidence interval	p-value
**Grip strength (kg)**	Unadjusted	-0.18	-0.51,0.15	0.28	-0.08	-0.19,0.04	0.18
	Adjusted for age, height, BMI	-0.18	-0.50,0.14	0.26	-0.06	-0.17,0.05	0.27
	Adjusted for height, BMI, smoking, education and SEP	-0.18	-0.54,0.17	0.30	-0.09	-0.21,0.02	0.12
**Chair rise speed (stands/minute)**	Unadjusted	-0.07	-0.40,0.26	0.67	-0.02	-0.13,0.09	0.75
	Adjusted for age, height, BMI	-0.07	-0.39,0.25	0.66	-0.01	-0.12,0.09	0.82
	Adjusted for height, BMI, smoking, education and SEP	-0.12	-0.45,0.22	0.49	-0.04	-0.15,0.07	0.45
**Balance time, eyes closed (log seconds)**	Unadjusted	-0.009	-0.03,0.01	0.42	-0.0002	-0.01,0.01	0.97
	Adjusted for age, height, BMI	-0.009	-0.03,0.01	0.41	0.001	-0.01,0.01	0.85
	Adjusted for height, BMI, smoking, education and SEP	-0.007	-0.03,0.02	0.53	-0.0005	-0.01,0.01	0.90
**Composite score**	Unadjusted	0.001	-0.004,0.007	0.67	0.001	-0.003,0.006	0.60
	Adjusted for age, height, BMI	0.002	-0.003,0.007	0.54	0.003	-0.002,0.007	0.22
	Adjusted for height, BMI, smoking, education and SEP	0.003	-0.002,0.009	0.25	0.002	-0.003,0.007	0.38

[2] For each of the four physical capability outcome measures, we ran three models, first unadjusted, then adjusted for height and BMI, then adjusted for height, BMI, smoking, education and both adult and childhood SEP

Table S3: Association of age acceleration with changes in physical capability from age 53 to 64 in NSHD participants, including those unable to perform tests

		Blood (n=152)			Buccal (n=790)		
**Variable**	Model[3]	Regression Coefficient (difference per year AA)	95% confidence interval	p-value	Regression Coefficient (difference per year AA)	95% confidence interval	p-value
**Grip strength (kg)**	Unadjusted	-0.30	-0.69,0.10	0.14	0.02	-0.14,0.17	0.83
	Adjusted for age, height, BMI	-0.28	-0.67,0.11	0.16	0.02	-0.13,0.17	0.80
	Adjusted for height, BMI, smoking, education and SEP	-0.29	-0.73,0.15	0.19	-0.04	-0.20,0.13	0.67
**Chair rise speed (stands/minute)**	Unadjusted	0.21	-0.23,0.64	0.35	-0.01	-0.16,0.14	0.87
	Adjusted for age, height, BMI	0.20	-0.23,0.63	0.36	-0.03	-0.18,0.12	0.74
	Adjusted for height, BMI, smoking, education and SEP	0.07	-0.40,0.54	0.77	-0.04	-0.20,0.12	0.62
**Balance time, eyes closed (log seconds)**	Unadjusted	-0.02	-0.05,0.01	0.21	-0.002	-0.01,0.01	0.77
	Adjusted for age, height, BMI	-0.02	-0.05,0.01	0.21	-0.003	-0.01,0.01	0.65
	Adjusted for height, BMI, smoking, education and SEP	-0.01	-0.05,0.02	0.44	-0.004	-0.02,0.01	0.48

[3] For each of the four physical capability outcome measures, we ran three models, first unadjusted, then adjusted for height and BMI, then adjusted for height, BMI, smoking, education and both adult and childhood SEP
