# Supplementary material for: Are objective measures of physical capability related to accelerated epigenetic age? Findings from a British birth cohort
Source: BMJ Open. 2017 Nov 1;7(10):e016708. doi: 10.1136/bmjopen-2017-016708 (PMC5695310; doi:10.1136/bmjopen-2017-016708)
Supplement: Author's manuscript [file bmjopen-2017-016708.draft_revisions.pdf]

BMJ Open

BMJ Open is committed to open peer review. As part of this commitment we make the peer review history of every article we publish publicly available.

When an article is published we post the peer reviewers' comments and the authors' responses online. We also post the versions of the paper that were used during peer review. These are the versions that the peer review comments apply to.

The versions of the paper that follow are the versions that were submitted during the peer review process. They are not the versions of record or the final published versions. They should not be cited or distributed as the published version of this manuscript.

BMJ Open is an open access journal and the full, final, typeset and author-corrected version of record of the manuscript is available on our site with no access controls, subscription charges or pay-per-view fees (http://bmjopen.bmj.com).

If you have any questions on BMJ Open's open peer review process please email info.bmjopen@bmj.com

Epigenetic age and objective measures of physical capability in the MRC National Survey of Health and Development

For peer review only

Journal:	*BMJ Open*
Manuscript ID	bmjopen-2017-016708
Article Type:	Research
Date Submitted by the Author:	24-Mar-2017

Complete List of Authors:	Simpkin, Andrew; MRC Integrative Epidemiology Unit, ; University of Bristol<br>Cooper, Rachel; UCL, MRC Unit for Lifelong Health and Ageing at UCL<br>Howe, Laura; University of Bristol, Social Medicine<br>Relton, Caroline; University of Bristol,<br>Davey Smith, George; University of Bristol, Social Medicine<br>Teschendorff, Andrew; UCL<br>Widschwendter, Martin; University College Hospital, 2Department of Women's Cancer<br>Wong, Andrew; UCL, MRC Unit for Lifelong Ageing<br>Kuh, Diana; MRC Unit for Lifelong Health and Ageing at UCL,<br>Hardy, Rebecca; University College London

**Primary Subject Heading**:	Epidemiology
Secondary Subject Heading:	Genetics and genomics
Keywords:	ALSPAC, DNA methylation, epigenetic age, NSHD, physical capability

SCHOLARONE™ Manuscripts

Epigenetic age and objective measures of physical capability in the MRC National Survey of Health and Development

Andrew J Simpkin[*1], Rachel Cooper[2], Laura D Howe[1], Caroline L Relton[1], George Davey Smith[1], Andrew Tescendorff[3,4,5], Martin Widschwendter[3], Andrew Wong[2], Diana Kuh[2], Rebecca Hardy[2]*

1. MRC Integrative Epidemiology Unit, School of Social and Community Medicine, University of Bristol, Bristol BS8 2PS, UK

2. MRC Unit for Lifelong Health and Ageing at UCL, London WC1B 5JU, UK

3. Department of Women's Cancer, University College London, Medical School Building, Room 340, 74 Huntley Street, London WC1E 6AU, UK

4. CAS Key Lab of Computational Biology, CAS-MPG Partner Institute for Computational Biology, Shanghai Institute for Biological Sciences, Chinese Academy of Sciences, Shanghai 200031, China

5. Statistical Cancer Genomics, Paul O'Gorman Building, UCL Cancer Institute, University College London, 72 Huntley Street, London WC1E 6BT, UK

* Corresponding author

Address:

Barley House

Oakfield Road

Clifton

Bristol BS8 2PS

United Kingdom

Email: andrew.simpkin@bristol.ac.uk

Phone: +44 117 33 14026

Fax: +44 117 33 10123

Word count: 3547

Keywords: ALSPAC, DNA methylation, epigenetic age, NSHD, physical capability

For peer review only - http://bmjopen.bmj.com/site/about/guidelines.xhtml

Abstract

Objectives: Our aim was to investigate the association of epigenetic age and physical capability in later life. Having a higher epigenetic than chronological age (known as age acceleration, AA) has been found to be associated with an increased rate of mortality. Similarly, physical capability has been proposed as a marker of ageing due to its consistent associations with mortality.

Setting: The MRC National Survey of Health and Development (NSHD)

Participants: We used data from 790 women from the NSHD who had DNA methylation data available.

Design: Epigenetic age was calculated using buccal cell (n=790) and matched blood tissue (n=152) from 790 female NSHD participants. We investigated the association of AA at age 53 with changes in physical capability in women from ages 53 to 60-64. Regression models of change in each measure of physical capability on AA were conducted. Secondary analysis focussed on the relationship between AA and smoking, alcohol, body mass index (BMI) and socioeconomic position.

Outcome measures: Three objective measures of physical capability were used: grip strength, standing balance time and chair rise speed.

Results: Epigenetic age was lower than chronological age (mean 53.4) for both blood (50.3) and buccal cells (42.8). AA from blood was associated with a greater decrease in grip strength from age 53 to 60-64 (0.42kg decrease per year of AA (0.03, 0.82kg decrease; p=0.03, n=152), but no associations were observed with standing balance time or chair rise speed. Current smoking and lower BMI were associated with lower epigenetic age from buccal cells.

Conclusions: We found evidence that AA in blood is associated with a greater decrease in grip strength but not with standing balance time or chair rise speed.

For peer review only - http://bmjopen.bmj.com/site/about/guidelines.xhtml

Strengths and limitations of this study

- Our study is one of the first to examine epigenetic age from different tissues on the same individuals in relation to objective measures of physical capability, which are key markers of healthy ageing.
- We used serial measures of physical capability on the same individuals over time, allowing for better inferences on changes in physical capability in late midlife, compared with having cross sectional data.
- A limitation of our findings is the lack of generalisability - the subsample of the cohort consisted of females only with repeated measures at ages 53 and 64 and was restricted to those with complete information on particular variables of interest (i.e. blood and buccal

review only

For peer review only - http://bmjopen.bmj.com/site/about/guidelines.xhtml

INTRODUCTION

There has been considerable recent interest in epigenetic biomarkers of ageing[1-6], which use an individual's DNA methylation data to estimate their "epigenetic age", a concept that could be considered a form of biological age. The Horvath age estimation method[1] found a correlation of 0.96 between chronological and epigenetic age, with individual estimates of epigenetic age within 3 years of chronological age on average. Epigenetic age has the potential to assess our biological age, but little is known about its relationship with our basic physiology. Moreover, several recent papers have found that the difference between epigenetic and chronological age (known as age acceleration, denoted AA) has biological significance. A positive AA indicates an individual's epigenetic age is ahead of their chronological age, a negative AA (i.e. age deceleration) suggests an individual has younger epigenetic age than chronological age. For example, positive AA has been found to be associated with obesity[7], Down's syndrome[8], HIV[9], menopause[10], and all-cause mortality[11 12].

Lower physical capability, assessed using objective measures such as grip strength, chair rise speed and standing balance time have been found to be associated with all-cause mortality[13]. These findings, established through a systematic review of mainly older populations, were also observed using data on physical capability in midlife from the MRC National Survey of Health and Development (NSHD)[14-16], which has followed 5362 individuals born in the same week of March 1946.

It is pertinent to understand the mechanisms underpinning the association between epigenetic age and mortality, since epigenetics are a potentially modifiable risk factor. A recent study identified a cross-sectional association between epigenetic age acceleration and lower grip strength in an older population using data from the Lothian Birth Cohort 1936[17]. There was no strong evidence for links between epigenetic age and changes in grip strength, lung function or cognition from age 70 to 76. In the present article we sought to investigate the associations between epigenetic age at age 53 and physical capability at ages 53 and 60-64 in the NSHD. We hypothesised that individuals with

For peer review only - http://bmjopen.bmj.com/site/about/guidelines.xhtml

positive age acceleration (i.e. with epigenetic age higher than expected based on a linear regression of epigenetic age on chronological age) would have lower average physical capability scores and greater declines than those with lower epigenetic age. We also use data from the NSHD to investigate whether increased epigenetic age is associated with known mortality risk factors; smoking, higher body mass index (BMI) and more disadvantaged socioeconomic position (SEP).

For peer review only

For peer review only - http://bmjopen.bmj.com/site/about/guidelines.xhtml

METHODS

Study participants

DNA was first collected in NSHD participants at age 53[18]; following QC, the study sample with epigenetic information available consisted of 790 women selected from those who had a buccal cell sample taken at age 53 (mean age 53.4, standard deviation 0.16, range 53 to 54). Among these 790 women there were 152 who also had epigenetic information available from blood at age 53. These 152 were originally selected for a study of epigenetics and cancer, and consisted of 75 incident cancer cases after age 53 and 77 controls [16 19 20]. Mortality risk factor data were available at age 53 on smoking status (current, never or ex-smoker), nurse measured height (cm) and weight (kg) (used to calculate BMI (kg/m^2)), and socio-economic position (SEP) in both childhood (father's occupational class) and adulthood (education and occupational class). Education level attained was classified as none, vocational, sub GCSE, O level, A level, degree or higher. Father's occupational class in childhood and own occupational class in adulthood were defined according to the Registrar General's social classification: unskilled, partly skilled, skilled (manual), skilled (non-manual), intermediate or professional.

Study outcomes

The three measures of physical capability were grip strength (kg), standing balance time (seconds) and chair rise time (seconds) measured at age 53 and again at age 60-64 by nurses using standardised protocols[21]. Grip strength was ascertained isometrically using an electronic dynamometer which was calibrated using a back-loading rig and was stable to within 0.5kg. Two values from each hand were recorded at 53, and three in each hand at 60-64, with the maximum of the first four values at each age used for analysis. The standing balance test recorded the times that participants could stand on one leg up to a maximum of 30 seconds first with eyes open and then repeated with eyes closed. Balance times with eyes closed were used for analysis and these were log transformed to reduce skewness. Chair rise time was measured using a stopwatch and recorded as

For peer review only - http://bmjopen.bmj.com/site/about/guidelines.xhtml

the time taken to rise from a seated position to a standing position with a straight back and legs followed by a return to a seated position as fast as possible, repeated 10 times. Chair rise speed was then calculated by dividing the number of rises (i.e. 10) by the time taken in minutes. This was done to make high scores correspond to good performance, as for the other two measures. Nurses recorded whether the participant was unwilling or unable to perform each of the tests along with the reason for this.

Composite capability scores were generated by combining performance on grip strength, balance time and chair rise speed using methods previously described[14]. In brief, each measure was rescaled to a 0 (low) – 1 (high) scale before aggregation into a composite score from 0-3 at ages 53 and 60-64. Standing balance time was rescaled by dividing by 30 seconds (the maximum time allowed); height adjusted grip strength and chair rise time were rescaled by dividing by the 99th percentile. Those unable to carry out a test for health reasons were assigned a score of 0 for that test.

DNA methylation data

DNA methylation was measured using the Infinium HumanMethylation450 BeadChip (Illumina, Inc) in a subsample of 790 female NSHD participants; 638 (buccal cell only) and 152 (buccal cell and blood) [19]. All participants provided written informed consent. The Central Manchester Ethics Committee approved the use of these samples for epigenetic studies of health.

Epigenetic age

Using the online epigenetic clock calculator (http://labs.genetics.ucla.edu/horvath/dnamage), we obtained DNA methylation estimated age using the Horvath[1] method. The raw DNA methylation β-values were generated from the 152 blood and 790 buccal cell samples. Along with epigenetic age, the online calculator estimates raw age acceleration differences (epigenetic-chronological age) and age acceleration residuals (the residuals from a linear regression of epigenetic age on chronological age). Our main exposure of interest is the latter age acceleration residual, which we will call age

For peer review only - http://bmjopen.bmj.com/site/about/guidelines.xhtml

acceleration and denote AA. AA values from blood were corrected for estimated cell type heterogeneity using the Houseman method[22].

Statistical analysis

Pearson correlation coefficients were used to investigate the relationship between chronological age and epigenetic age from blood and buccal tissue. Change in each physical capability measure was considered the main outcome, with the difference in grip strength, chair rise speed, balance time and composite score from age 53 to age 60-64 being used for analysis. Using this unconditional change model allows us to directly compare our results with those from the Lothian Birth Cohort 1936[17]. The differences were regressed on AA from blood and buccal tissue separately. We fitted unadjusted regression models followed by models adjusted for age, height and BMI and then additionally adjusted for smoking and both childhood and adult SEP. Linear regression was also used to test the association of AA (from both blood and buccal cells) at age 53 with each physical capability measure and the composite score at both ages 53 and 60-64. As a secondary analysis with AA as the outcome, we carried out unadjusted regression analysis of the known mortality risk factors of height, BMI, smoking and SEP (both childhood and adult).

For each of the three measures of physical capability, those who were unable to perform each task for health reasons (Table 1 includes percentage unable to perform each task) were allocated the minimum sex-specific value. We include these imputed data in a sensitivity analysis.

Replication

Findings were tested for replication using cross sectional data from the mothers of the Avon Longitudinal Study of Parents and Children (ALSPAC)[23 24]. ALSPAC recruited 14541 pregnant women with expected delivery dates between April 1991 and December 1992. Of these initial pregnancies there were 14062 live births and 13988 children who were alive at one year of age. The study website contains details of all the data that are available through a fully searchable data dictionary

For peer review only - http://bmjopen.bmj.com/site/about/guidelines.xhtml

(http://www.bris.ac.uk/alspac/researchers/data-access/data-dictionary). DNA methylation and epigenetic age were available from 988 ALSPAC mothers at mean age of 46.9 (standard deviation 4.7 years, range 31 to 60) as part of the Accessible Resource for Integrated Epigenetics Studies (ARIES) project[25]. Grip strength, balance time and chair rise speed along with height, BMI, smoking and SEP (adulthood only) were available from these same women. Grip strength was assessed using the Jamar handgrip dynamometer and was recorded to the nearest 1kg using both the right and left hands. Two measures were taken in each hand and the maximum of these values was used. In the chair rise test, the participant was asked to rise from a sitting position to a straight-legged fully standing position five times while being timed. Chair rise speed was then calculated by dividing five by the total time required. This differs from NSHD in having five total stands, though most of the between study variability would be resolved by using chair rise speed rather than total time. In the balance time test, the participant stood next to a table and asked to choose a leg and raise it off the floor to ankle height. The participant was timed until they lost their balance and drop their foot or had to reach out to the table for support. If the participant remained on one leg for longer than 30 seconds they were stopped. The process was repeated with eyes closed, which was used for analysis to mirror the NSHD measure.

For peer review only

For peer review only - http://bmjopen.bmj.com/site/about/guidelines.xhtml

RESULTS

The descriptive statistics are displayed in Table 1, with epigenetic ages shown in Figures 1. The average epigenetic age was 42.8 (SD 5.71 years) using DNA methylation from buccal tissue and 50.3 (SD 4.34 years) using DNA methylation from blood. These both underestimate the average chronological age of 53.4 years (SD 0.16 years). Correlation with chronological age was much lower than previously reported: 0.022 (p=0.79) for blood age and 0.115 (p=0.16) for buccal age. Correlation was slightly higher between the two epigenetic ages, with a Pearson correlation coefficient of 0.190 (p=0.02).

Age acceleration (AA), being the residual of a regression of epigenetic age on chronological age, has a mean close to zero by definition. However the variance and range are larger for AA of buccal tissue compared to blood tissue. Average levels of each physical capability measure changed in the expected direction, with a mean decrease in grip strength of 2.6kg (SD 8.5kg), chair rise speed of 6.8 stands/minute (SD 10.1 stands/min) and balance time of 0.27 log-seconds (SD 0.68 log secs) from age 53 to 60-64. This is reflected in an average decrease of 0.12 units in the composite score for physical capability.

Age acceleration and physical capability

<u>Change in physical capability</u>

For a 1-year increase in AA, grip strength decreased by an additional 0.42kg (95% CI 0.03, 0.82kg; p=0.03) from age 53 to 60-64 after adjusting for height, BMI, education and SEP (Table 2). Surprisingly, the association of AA from blood and change in chair rise speed was positive, with a 1-year AA being associated with). There was no strong evidence for an association between AA and change in chair rise speed (0.06 higher stands per minute per 1-year AA 95% CI -0.40, 0.52 stands per minute; p=0.80) or balance time (0.01 log-seconds lower per 1-year AA, 95% CI -0.04, 0.02 log-

For peer review only - http://bmjopen.bmj.com/site/about/guidelines.xhtml

seconds; p=0.55). The weak associations of AA from buccal cells and physical capability were in the expected direction.

Separate analysis of physical capability measured at 53 and 60-64

There were no associations between physical capability at age 53 and epigenetic age, either from blood or buccal samples (Table S1). Effect sizes were much smaller in buccal AA compared with blood AA for grip strength and particularly for chair rise speed. The associations of AA with grip strength, balance time and the composite score were positive, indicating greater AA was associated with better performance, i.e. the opposite direction to that expected. Similarly, there was little evidence for an association between epigenetic age and any of the physical capability markers or the composite score at age 60-64 (Table S2). Here the effect of AA was in the expected negative direction for grip strength, chair rise speed and balance time, with stronger effects observed from blood AA than buccal AA.

Age acceleration and mortality risk factors

There were positive associations between BMI at age 53 and AA from buccal tissue: 0.085 (95% CI: 0.014, 0.156; p=0.02) years of AA per 1kg/m^2 increase in BMI. The strength of association was lower for blood tissue: 0.044 years of AA per 1kg/m^2 change in BMI (95% CI: -0.065, 0.154 years; p=0.42) (Table 3). There was no association between height and AA. We observed an association between smoking and AA of buccal tissue (p=0.001), but not in blood tissue. Current smokers had the lowest AA on average, with ex- and never-smokers having 1.88 (95% CI 0.85, 2.9) and 1.85 (95% CI 0.76, 3.0) extra years of AA. AA did not vary by childhood or adult SEP.

Sensitivity analysis

We provide associations of AA and change in physical capability when including imputed data for those unable to perform tests in Table S3. Including individuals unable to perform the grip strength test attenuates its association with AA. For a 1-year increase in AA, grip strength decreased by an

For peer review only - http://bmjopen.bmj.com/site/about/guidelines.xhtml

For peer review only

additional 0.29kg (95% CI -0.74, 0.15kg; p=0.19) from age 53 to 60-64 in a fully adjusted model. Including individuals unable to perform tasks did not dramatically affect any of the other blood AA or buccal associations.

Replication

Using data from 988 ALSPAC women with mean age 46.9, we attempted to replicate the cross-sectional findings from NSHD (Tables 4 and 5). AA was not related to grip strength, chair rise speed or balance time. The finding that higher BMI was associated with greater AA was replicated in the ALSPAC women (0.129 years per 1kg/m^2 increase in BMI, 95% CI 0.051, 0.207 years, p=0.001) but smoking was not associated with AA (p=0.43), although the direction of effect was the same, with ex-smokers having 0.56 years higher AA and never smokers 0.17 years higher AA compared to current smokers on average. As in NSHD, height and education were not associated with AA.

For peer review only - http://bmjopen.bmj.com/site/about/guidelines.xhtml

For peer review only

DISCUSSION

Age acceleration in blood is associated with a greater decline in grip strength from age 53 to 60-64; for every one-year of AA, women had a 0.4kg greater decrease in grip strength. Neither blood nor buccal epigenetic age at age 53 was associated with grip strength or other measures of physical capability at either 53 or 60-64. The epigenetic age calculated in our sample was systematically lower than chronological age, particularly for buccal cells (mean difference of 10.7 years for buccal cells).

Our study is one of the first to examine epigenetic age from different tissues on the same individuals in relation to risk factors for mortality. This has allowed discussion around tissue specificity in the growing research based on epigenetic age. We used serial measures of physical capability on the same individuals over time, allowing for better inferences on changes in physical capability in late midlife, compared with having just cross sectional data. However, one limitation is having just two measures, which are susceptible to regression to the mean. A limitation of our findings is the lack of generalisability - the subsample of the cohort consisted of females only with repeated measures at ages 53 and 64 and was restricted to those with complete information on particular variables of interest (i.e. blood and buccal samples). The 790 women sampled here had marginally lower grip strength (25.7kg vs 26.0kg, p=0.4) and chair rise speed (24.5 stands/min vs 25.5 stands/min, p=0.007) than NSHD women overall at age 60-64[26]. Including those individuals who were unable to perform the grip strength test attenuated the association with blood AA. However, although 18 and 21 grip strength tests could not be performed at 53 and 60-64 respectively (Table 1), just six of these were from individuals with blood DNA methylation available. The attenuation was mainly due to a single individual with low AA and high grip strength at age 53 who was unable to perform the test at age 60-64.

Our blood results should be compared to another recent study of epigenetic age and physical capability in an older UK birth cohort[17]. Using data from the Lothian Birth Cohort 1936, cross

For peer review only - http://bmjopen.bmj.com/site/about/guidelines.xhtml

sectional associations at age 70 were found between greater epigenetic age and weaker grip strength as well as with lower lung function and cognitive capability, but not walking speed; however they found no association between baseline epigenetic age and changes in either physical or cognitive capability from age 70 to 76. We have found some evidence that epigenetic age measured at 53 may be associated with a greater decline in grip strength between 53 and 60-64, but no associations were identified with any physical capability measure at 53 or 60-64. In the Lothian Birth Cohort, the reported effect size of AA on grip strength at age 70 was -0.05 per year of blood AA with a sample size of 1004[17]. In our analysis the effect sizes at 53 and 60-64 were 0.18kg and -0.15kg per year of blood AA respectively with a sample size of 152. The relatively small sample in our analysis of blood AA may mean our study lacks the required power, or it could be that this association only manifests at older ages. It is possible that the association is beginning to emerge in NSHD at 60-64 (an age still younger than the Lothian Birth Cohort baseline) with the suggestion of faster rates of change in those with greater AA. We do not, however, observe any associations between buccal cell AA and physical capability where we have a larger sample size and more comparable statistical power to the Lothian Birth Cohort. Our different results could also be due to sex differences between the two studies; the LBC includes both men and women, whereas we have only looked in females. Several studies have identified higher AA in men than in women[27]. To better understand the epigenetic embodiment of physical capability in later life, one might perform epigenome wide analysis of these measures.

We found a weak correlation between epigenetic age from blood and buccal cells in the same individuals (r=0.19). While the very small range of age at which the epigenetic information was taken in our sample (standard deviation of 0.16 years, range 53-54 years) may explain the low correlation between epigenetic and chronological ages (0.022 for blood, 0.115 for buccal cells), it does not explain the bias. The systematic difference found here appears to be related to tissue specificity, since epigenetic age from blood was closer to chronological age than buccal epigenetic age. The Horvath age estimator was developed using publicly available data covering 51 tissue types

For peer review only - http://bmjopen.bmj.com/site/about/guidelines.xhtml

(including buccal cells) such that tissue specificity should not result in such an underestimate of chronological age. Three sets of publicly available buccal cell DNA methylation data[28-30] were among those used in the development of the Horvath epigenetic clock, with a reported correlation of 0.9 between chronological and buccal epigenetic age[1]. However these were from 109 adolescents[28], 30 newborns (i.e. ten pairs of MZ and five pairs of DZ twins between birth and 18 months[30]) and ten individuals who were aged 16, 27, 28, 29, 37, 42, 44, 44, 52, 68[29]. The systematic difference (of 10.7 years) may be explained by the lack of overlap in the age at which information from buccal cells was available in our study and those in the training dataset used to derive the epigenetic clock. There are a growing number of studies comparing DNA methylation from more than one tissue on the same group of individuals [10 19 31], with one of these using the same NSHD data as the current study[10]. One novel application of the epigenetic clock is to estimate epigenetic ages from different tissues on the same individuals. In the current study we have found evidence that buccal samples are epigenetically younger than blood samples in a UK population. Further comparisons of epigenetic ages from different tissue types in the same individuals may elucidate our findings.

We found both lower BMI and smoking were related to age deceleration, with the BMI finding replicated in blood methylation age from ALSPAC women at mean age 46.9. Previous research has found this same association between higher BMI and AA in liver tissue[7 27], but ours is the first finding in buccal cells. Our finding that smoking is associated with lower AA is unexpected, since previous research suggests that positive blood AA is associated with higher rates of mortality[11]. This could be due to our use of buccal cells, which are likely more reflective of the effect of smoking on DNA methylation[19]. It is currently unknown whether buccal AA is associated with mortality, nor if the direction is the same as blood AA. Further to this point, the smoking and AA association was not seen in blood samples from ALSPAC or NSHD participants, suggesting this particular result may be spurious.

For peer review only - http://bmjopen.bmj.com/site/about/guidelines.xhtml

In conclusion, having a higher epigenetic than chronological age is associated with a greater decline in grip strength in middle age, but overall there is little evidence that AA is associated with physical capability change in middle aged women. AA does not appear to be related to measures of physical capability in women at ages 53 or 60- 64, while BMI appears to be associated with accelerated epigenetic age in this population.

For peer review only

For peer review only - http://bmjopen.bmj.com/site/about/guidelines.xhtml

FUNDING

This work was supported by a grant from the UK Economic and Social Research Council (ES/M010317/1) and by the UK Medical Research Council (programme codes MC_UU_12019/1, MC_UU_12019/2, MC_UU_12019/4). Research reported in this publication was supported by the National Institute on Ageing of the National Institutes of Health under Award No. R01AG048835. AJS was supported by the UK Medical Research Council (grant MR/L011824/1). LDH was supported by a fellowship from the UK Medical Research Council (MR/M020894/1). The UK Medical Research Council and the Wellcome Trust (Grant ref: 102215/2/13/2) and the University of Bristol provide core support for ALSPAC. Collection of ALSPAC epigenetic and phenotypic data were funded by grants from the Wellcome Trust (WT092830M) and Joint UK Research Councils under the *Lifelong Health and Wellbeing - Phase 3 initiative* (G1001357).

ACKNOWLEDGEMENTS

For use of the ALSPAC data, we are extremely grateful to all the families who took part in this study, the midwives for their help in recruiting them, and the whole ALSPAC team, which includes interviewers, computer and laboratory technicians, clerical workers, research scientists, volunteers, managers, receptionists and nurses. Ethical approval for ALSPAC was obtained from the ALSPAC Ethics and Law Committee and the Local Research Ethics Committees. The content is solely the responsibility of the authors and does not necessarily represent the official views of the National Institutes of Health.

CONTRIBUTORSHIP STATEMENT

This publication is the work of the authors and Andrew Simpkin will serve as guarantors for the contents of this paper. AJS performed designed and ran analysis, drafted the manuscript. RC, RH, DK, AW, AT, MW supervised NSHD analysis. GDS, CLR, LDH supervised ALSPAC analysis. All authors contributed to writing the manuscript.

For peer review only - http://bmjopen.bmj.com/site/about/guidelines.xhtml

DATA SHARING STATEMENT

Data used in this publication are available to bona fide researchers upon request to the NSHD Data Sharing Committee via a standard application procedure. Further details can be found at: http://www.nshd.mrc.ac.uk/data; doi: 10.5522/NSHD/Q101; doi: 10.5522/NSHD/Q102.

COMPETING INTERESTS

None declared

REFERENCES

1. Horvath S. DNA methylation age of human tissues and cell types. *Genome Biol* 2013;14(10):R115.
2. Hannum G, Guinney J, Zhao L, et al. Genome-wide methylation profiles reveal quantitative views of human aging rates. *Mol Cell* 2013;49(2):359-67.
3. Bocklandt S, Lin W, Sehl ME, et al. Epigenetic predictor of age. *PLoS One* 2011;6(6):e14821.
4. Jones MJ, Goodman SJ, Kobor MS. DNA methylation and healthy human aging. *Aging cell* 2015:DOI:10.1111/acel.12349.
5. Weidner CI, Lin Q, Koch CM, et al. Aging of blood can be tracked by DNA methylation changes at just three CpG sites. *Genome Biol* 2014;15(2):R24.
6. Zbieć-Piekarska R, Spólnicka M, Kupiec T, et al. Examination of DNA methylation status of the ELOVL2 marker may be useful for human age prediction in forensic science. *Forensic Science International: Genetics* 2015;14:161-67.
7. Horvath S, Erhart W, Brosch M, et al. Obesity accelerates epigenetic aging of human liver. *Proceedings of the National Academy of Sciences* 2014;111(43):15538-43.
8. Horvath S, Garagnani P, Bacalini MG, et al. Accelerated epigenetic aging in Down syndrome. *Aging cell* 2015;14(3):491-95.
9. Horvath S, Levine AJ. HIV-1 infection accelerates age according to the epigenetic clock. *J Infect Dis* 2015:jiv277.
10. Levine ME, Lu AT, Chen BH, et al. Menopause accelerates biological aging. *Proceedings of the National Academy of Sciences* 2016:201604558.
11. Marioni RE, Shah S, McRae AF, et al. DNA methylation age of blood predicts all-cause mortality in later life. *Genome Biol* 2015;16(1):25.
12. Chen BH, Marioni RE, Colicino E, et al. DNA methylation-based measures of biological age: meta-analysis predicting time to death. *Aging (Albany NY)* 2016;8(9):1844.
13. Cooper R, Kuh D, Hardy R. Objectively measured physical capability levels and mortality: systematic review and meta-analysis. *BMJ* 2010;341:c4467.
14. Cooper R, Strand BH, Hardy R, et al. Physical capability in mid-life and survival over 13 years of follow-up: British birth cohort study. *BMJ: British Medical Journal* 2014;348 doi: 10.1136/bmj.g2219
15. Wadsworth M, Kuh D, Richards M, et al. Cohort profile: the 1946 national birth cohort (MRC National Survey of Health and Development). *Int J Epidemiol* 2006;35(1):49-54.
16. Kuh D, Pierce M, Adams J, et al. Cohort profile: updating the cohort profile for the MRC National Survey of Health and Development: a new clinic-based data collection for ageing research. *Int J Epidemiol* 2011;40(1):e1-e9.

For peer review only - http://bmjopen.bmj.com/site/about/guidelines.xhtml

For peer review only

17. Marioni RE, Shah S, McRae AF, et al. The epigenetic clock is correlated with physical and cognitive fitness in the Lothian Birth Cohort 1936. *Int J Epidemiol* 2015:dyu277.
18. Rousseau K, Vinall L, Butterworth S, et al. MUC7 haplotype analysis: results from a longitudinal birth cohort support protective effect of the MUC7* 5 allele on respiratory function. *Ann Hum Genet* 2006;70(4):417-27.
19. Teschendorff AE, Yang Z, Wong A, et al. Correlation of Smoking-Associated DNA Methylation Changes in Buccal Cells With DNA Methylation Changes in Epithelial Cancer. *JAMA oncology* 2015;1(4):476-85. doi: 10.1001/jamaoncol.2015.1053 [published Online First: 2015/07/17]
20. Anjum S, Fourkala E-O, Zikan M, et al. A BRCA1-mutation associated DNA methylation signature in blood cells predicts sporadic breast cancer incidence and survival. *Genome Med* 2014;6(6):47.
21. Kuh D, Bassey EJ, Butterworth S, et al. Grip strength, postural control, and functional leg power in a representative cohort of British men and women: associations with physical activity, health status, and socioeconomic conditions. *The Journals of Gerontology Series A: Biological Sciences and Medical Sciences* 2005;60(2):224-31.
22. Houseman EA, Accomando WP, Koestler DC, et al. DNA methylation arrays as surrogate measures of cell mixture distribution. *BMC Bioinformatics* 2012;13(1):86.
23. Fraser A, Macdonald-Wallis C, Tilling K, et al. Cohort profile: the Avon Longitudinal Study of Parents and Children: ALSPAC mothers cohort. *Int J Epidemiol* 2013;42(1):97-110.
24. Boyd A, Golding J, Macleod J, et al. Cohort profile: the 'children of the 90s'—the index offspring of the Avon Longitudinal Study of Parents and Children. *Int J Epidemiol* 2013;42:111-27.
25. Relton CL, Gaunt T, McArdle W, et al. Data Resource Profile: Accessible Resource for Integrated Epigenomic Studies (ARIES). *Int J Epidemiol* 2015:dyv072.
26. Cooper R, Muniz-Terrera G, Kuh D. Associations of behavioural risk factors and health status with changes in physical capability over 10 years of follow-up: the MRC National Survey of Health and Development. *BMJ open* 2016;6(4):e009962.
27. Simpkin AJ, Hemani G, Suderman M, et al. Prenatal and early life influences on epigenetic age in children: A study of mother-offspring pairs from two cohort studies. *Hum Mol Genet* 2015:ddv456.
28. Essex MJ, Thomas Boyce W, Hertzman C, et al. Epigenetic vestiges of early developmental adversity: childhood stress exposure and DNA methylation in adolescence. *Child Dev* 2013;84(1):58-75.
29. Rakyan VK, Down TA, Maslau S, et al. Human aging-associated DNA hypermethylation occurs preferentially at bivalent chromatin domains. *Genome research* 2010;20(4):434-39.
30. Martino D, Loke YJ, Gordon L, et al. Longitudinal, genome-scale analysis of DNA methylation in twins from birth to 18 months of age reveals rapid epigenetic change in early life and pair-specific effects of discordance. *Genome Biol* 2013;14(5):R42.
31. Davies MN, Volta M, Pidsley R, et al. Functional annotation of the human brain methylome identifies tissue-specific epigenetic variation across brain and blood. *Genome Biol* 2012;13(6):R43.

For peer review only - http://bmjopen.bmj.com/site/about/guidelines.xhtml

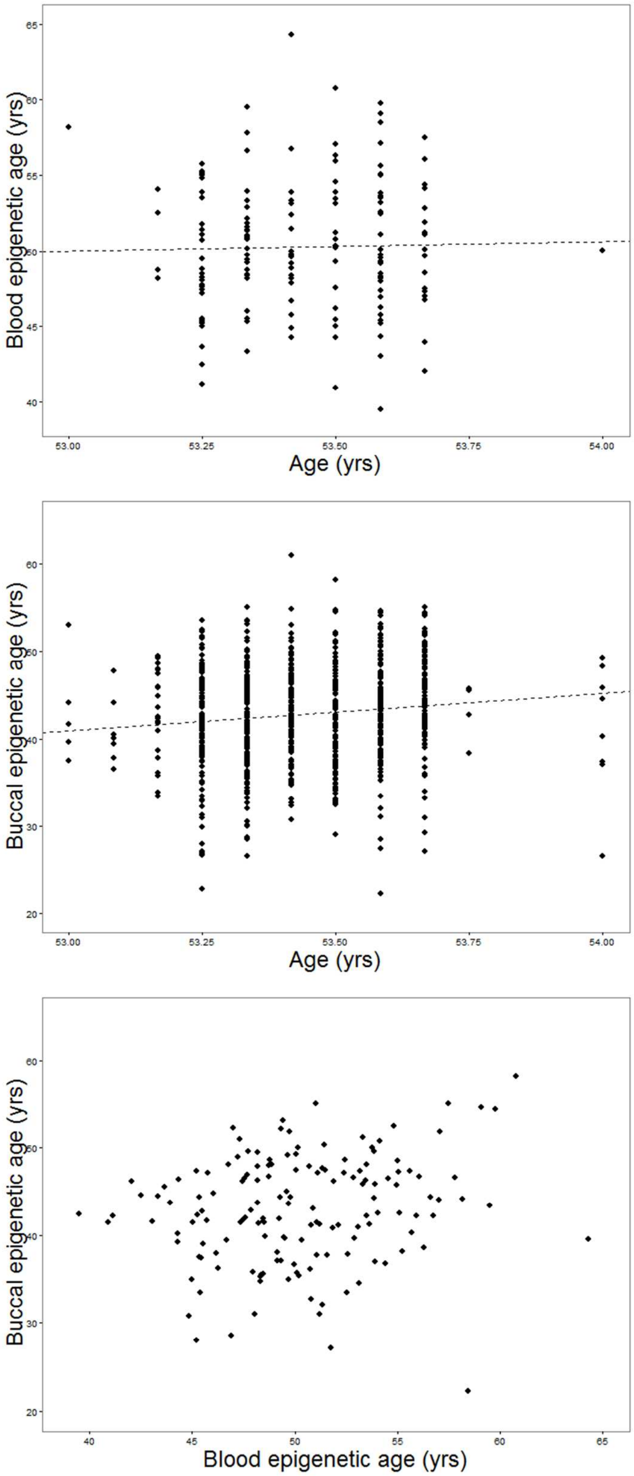

Figure 1: Blood age v chronological age, buccal age v chronological age and buccal age v blood age in NSHD, with line of best fit (dashed) to highlight age acceleration (above the line) and deceleration (below the line)

For peer review only - http://bmjopen.bmj.com/site/about/guidelines.xhtml

Table 1: Descriptive statistics for continuous variables

Continuous variable	**Age when measured**	**Mean (SD)**	**N**	**N unable (%)**
**Age (years)**	53	53.44 (0.16)	790	-
	64	63.09 (1.09)	623	-
**Epigenetic age from buccal (years)**	53	42.83 (5.71)	790	-
**Age acceleration from buccal (years)**	53	0.00 (5.66)	790	-
**Epigenetic age from blood (years)**	53	50.28 (4.34)	152	-
**Age acceleration from blood (years)**	53	0.00 (4.34)	152	-
**Height (cm)**	53	161.43 (5.61)	790	-
**BMI (kg/m2)**	53	28.13 (6.43)	784	-
	64	29.10 (7.50)	622	-
**Grip strength (kg)**	53	28.18 (8.15)	767	18 (2)
	64	25.71 (7.94)	575	21 (3)
**Grip strength change (kg)**	64	-2.62 (8.53)	560	
**Chair rise speed (stands/minute)**	53	30.98 (9.56)	737	40 (5)
	64	24.54 (7.82)	577	40 (5)
**Chair rise speed change (stands/minute**	64	-6.87 (10.07)	556	
**Balance time (log seconds)**	53	1.77 (0.60)	734	32 (8)
	64	1.52 (0.55)	593	24 (3)
**Balance time change (log seconds)**	64	-0.27 (0.68)	561	-
**Composite score**	53	1.31 (0.33)	750	-
	64	1.19 (0.37)	591	-
**Composite score change**	64	-0.13 (0.36)	558	-
Categorical variable	**Age when measured**	**Category**	**N (%)**	
**Smoking**	53	*Never*	384 (49)	
		Ex	239 (30)	
		Current	167 (21)	
**Childhood SEP**	53	*Professional*	57 (7)	
		Intermediate	160 (21)	
		Skilled (non-manual)	123 (16)	
		Skilled (manual)	245 (31)	
		Partly skilled	148 (19)	
		Unskilled	48 (6)	
**Adult SEP**	53	*Professional*	14 (2)	
		Intermediate	261 (33)	

For peer review only - http://bmjopen.bmj.com/site/about/guidelines.xhtml

		Skilled (non-manual)	286 (36)
		Skilled (manual)	57 (7)
		Partly skilled	119 (15)
		Unskilled	50 (7)
**Education**	53	*None*	275 (35)
		Vocational	44 (5)
		Sub GCE	38 (5)
		O level	201 (26)
		A level	106 (14)
		Burham A2	75 (10)
		Degree	32 (4)
		Postgrad	2 (1)

For peer review only

Table 2: Association of age acceleration with changes in physical capability from age 53 to 64 in NSHD participants

		**Blood (n=152)**			**Buccal (n=790)**		
**Variable**	Model[1]	Regression Coefficient (difference per year AA)	95% confidence interval	p-value	Regression Coefficient (difference per year AA)	95% confidence interval	p-value
**Grip strength (kg)**	Unadjusted	-0.34	-0.70,0.01	0.06	-0.02	-0.16,0.12	0.73
	Adjusted for age, height, BMI	-0.33	-0.69,0.02	0.06	-0.03	-0.17,0.12	0.73
	Adjusted for height, BMI, smoking, education and SEP	-0.42	-0.82,-0.03	0.03	-0.07	-0.22,0.08	0.35
**Chair rise speed (stands/minute)**	Unadjusted	0.20	-0.23,0.62	0.37	-0.03	-0.17,0.12	0.70
	Adjusted for age, height, BMI	0.19	-0.24,0.62	0.38	-0.04	-0.19,0.10	0.58
	Adjusted for height, BMI, smoking, education and SEP	0.06	-0.40,0.52	0.80	-0.05	-0.20,0.10	0.53
**Balance time, eyes closed (log seconds)**	Unadjusted	-0.01	-0.04,0.02	0.38	-0.001	-0.011,0.010	0.92
	Adjusted for age, height, BMI	-0.01	-0.04,0.02	0.39	-0.002	-0.012,0.009	0.76
	Adjusted for height, BMI, smoking, education and SEP	-0.01	-0.04,0.02	0.55	-0.002	-0.012,0.009	0.73
**Composite score**	Unadjusted	-0.01	-0.028,0.003	0.10	0.001	-0.005,0.007	0.77
	Adjusted for age, height, BMI	-0.01	-0.028,0.003	0.11	0.000	-0.005,0.006	0.87
	Adjusted for height, BMI, smoking, education and SEP	-0.01	-0.03,0.01	0.16	-0.001	-0.007,0.005	0.68

[1] For each of the four physical capability outcome measures, we ran three models, first unadjusted, then adjusted for height and BMI, then adjusted for height, BMI, smoking, education and both adult and childhood SEP

For peer review only - http://bmjopen.bmj.com/site/about/guidelines.xhtml

Table 3: Associations of mortality risk factors with outcome of age acceleration (years) at 53 for NSHD participants

		**Blood (n=152)**			**Buccal (n=790)**		
**Variable**	Level	Regression Coefficient (difference per year AA)	95% confidence interval	p-value	Regression Coefficient (difference per year AA)	95% confidence interval	p-value
**Height (cm)**		-0.017	-0.085,0.051	0.63	-0.011	-0.14,0.11	0.86
**BMI 53 (kg/m^2)**		0.085	0.014,0.16	0.02	0.044	-0.065,0.15	0.42
**Smoking**	Current	Reference		0.001	Reference		0.42
	Ex-smoker	1.88	0.85,2.91		0.83	-0.99,2.66	
	Never	1.86	0.76,2.95		-0.16	-2.17,1.85	
**Childhood SEP**	Professional	Reference		0.80	Reference		0.56
	Intermediate	-0.73	-2.44,0.99		-1.30	-4.01,1.40	
	Skilled (non-manual)	-0.81	-2.59,0.97		-0.48	-3.24,2.27	
	Skilled (manual)	-0.25	-1.89,1.38		-1.24	-3.88,1.39	
	Partly skilled	-0.94	-2.67,0.79		-0.11	-2.90,2.67	
	Unskilled	-0.40	-2.57,1.78		1.39	-2.40,5.17	
**Adult SEP**	Professional	Reference		0.62	Reference		0.35
	Intermediate	0.95	-2.11,4.01		-1.65	-10.27,6.97	
	Skilled (non-manual)	0.95	-2.10,4.00		-1.95	-10.59,6.69	
	Skilled (manual)	1.35	-1.97,4.68		-0.25	-9.13,8.63	
	Partly skilled	0.032	-3.12,3.18		-3.41	-12.31,5.50	
	Unskilled	0.52	-2.85,3.89		0.24	-8.73,9.22	
**Education**	None	Reference		0.78	Reference		0.64
	Vocational	0.16	-1.65,1.98		-1.25	-4.72,2.22	
	Sub GCE	1.20	-0.73,3.14		-1.74	-5.21,1.73	
	O level	0.41	-0.63,1.45		-1.02	-2.90,0.86	
	A level	-0.53	-1.81,0.75		-1.97	-4.29,0.35	
	Burham A2	0.55	-0.91,2.00		-1.43	-3.66,0.81	
	Degree	-0.28	-2.36,1.81		-2.01	-5.73,1.71	
	Postgrad	-0.81	-8.73,7.11		0.84	-0.38,2.06	

For peer review only - http://bmjopen.bmj.com/site/about/guidelines.xhtml

Table 4: Replication of physical capability and age acceleration in ALSPAC mothers with mean age 46.9

Variable	Model[2]	Regression coefficient (difference per year AA)	95% confidence interval	p-value
**Grip strength (kg)**	Unadjusted	-0.021	-0.12,0.078	0.68
	Adjusted for height, BMI	-0.044	-0.14,0.052	0.37
	Adjusted for height, BMI, SEP	-0.034	-0.13,0.065	0.50
**Chair rise speed (stands/minute)**	Unadjusted	0.0004	-0.0004,0.001	0.31
	Adjusted for height, BMI	0.0005	-0.0003,0.001	0.22
	Adjusted for height, BMI, SEP	0.0004	-0.0004,0.001	0.31
**Balance time, eyes closed (log seconds)**	Unadjusted	0.067	-0.058,0.19	0.30
	Adjusted for height, BMI	0.083	-0.045,0.21	0.20
	Adjusted for height, BMI, SEP	0.088	-0.042,0.22	0.18

Table 5: Replication of mortality risk factors with outcome of age acceleration in ALSPAC mothers with mean age 46.9

Variable	Level	Association with AA (years)	95% confidence interval	p-value
**Height (cm)**		0.0288	-0.034,0.092	0.37
**BMI (kg/m^2)**		0.1293	0.051,0.21	0.001
**Smoking**	Current	Reference		0.43
	Ex	0.5605	-0.88,2.00	
	Never	0.1693	-1.90,2.24	
**Education**	Secondary	Reference		0.28
	Vocational	0.1693	-1.90,2.24	
	O level	0.2586	-1.34,1.86	
	A level	-0.0295	-1.65,1.59	
	Degree	1.2266	-0.47,2.93	

[2] For each of the four physical capability measures, we ran three models, first unadjusted, then adjusted for height and BMI, then adjusted for height, BMI, smoking and education

For peer review only - http://bmjopen.bmj.com/site/about/guidelines.xhtml

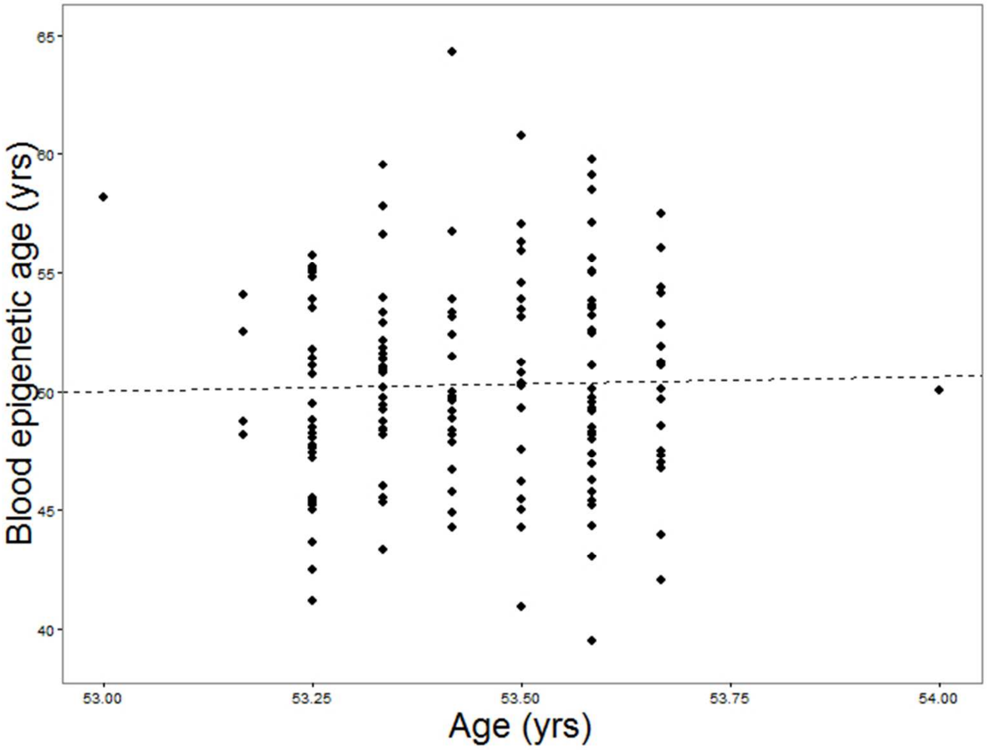

Blood age v chronological age, buccal age v chronological age and buccal age v blood age in NSHD, with line of best fit (dashed) to highlight age acceleration (above the line) and deceleration (below the line)

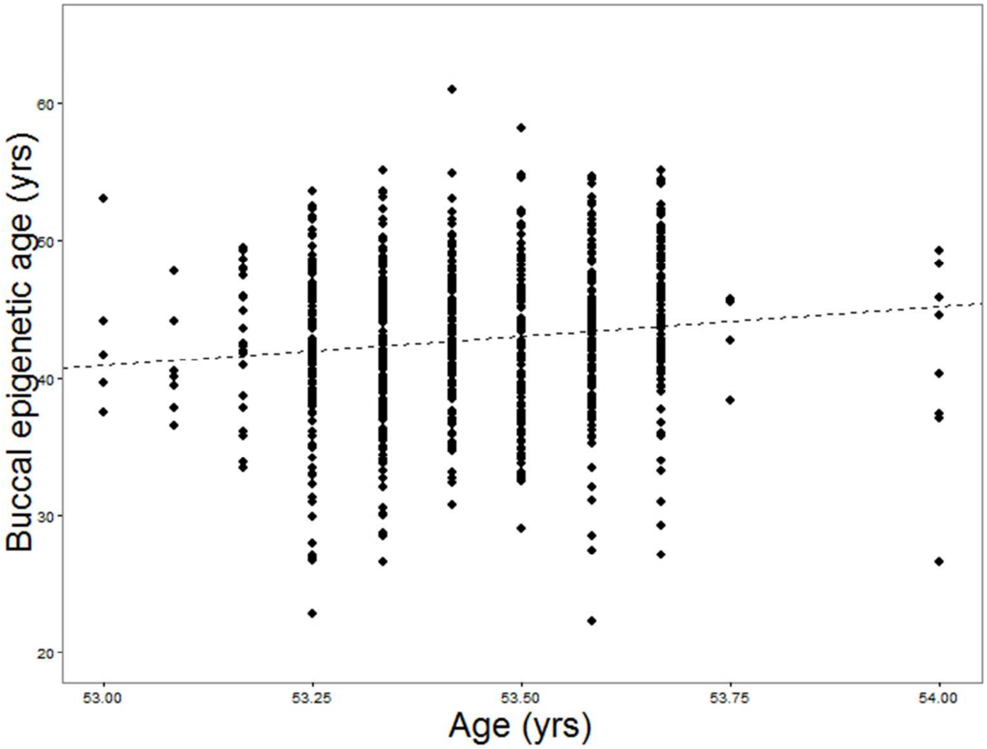

Blood age v chronological age, buccal age v chronological age and buccal age v blood age in NSHD, with line of best fit (dashed) to highlight age acceleration (above the line) and deceleration (below the line)

ew only

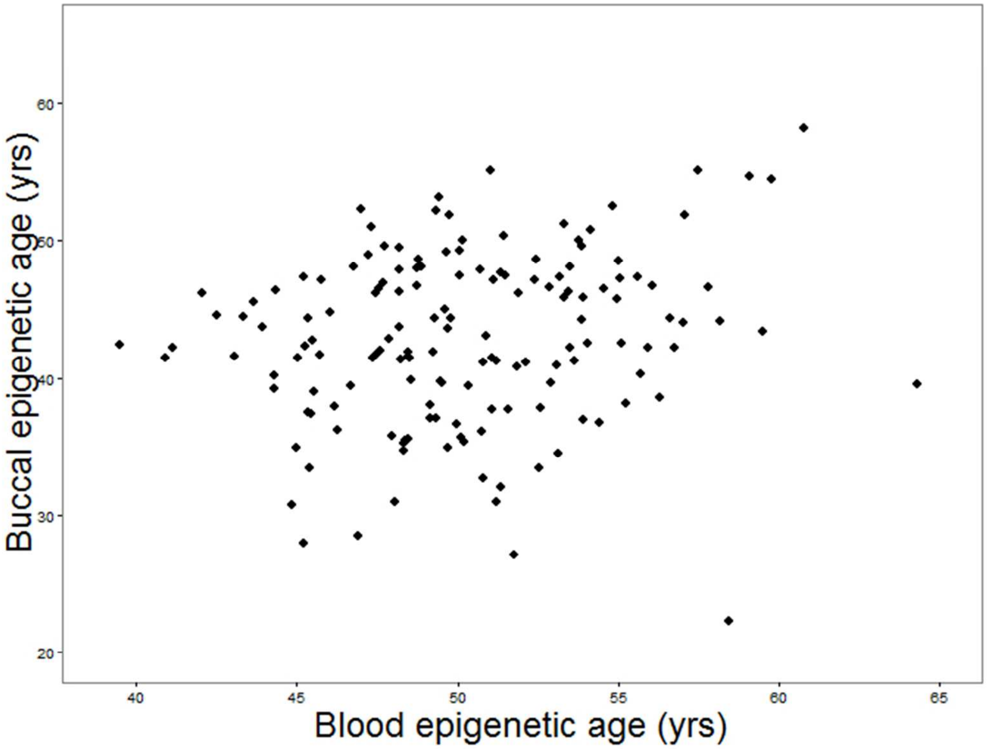

Blood age v chronological age, buccal age v chronological age and buccal age v blood age in NSHD, with line of best fit (dashed) to highlight age acceleration (above the line) and deceleration (below the line)

For peer review only - http://bmjopen.bmj.com/site/about/guidelines.xhtml

STROBE Statement—Checklist of items that should be included in reports of ***cohort studies***

	Item No	Recommendation	Page No
**Title and abstract**	1	(*a*) Indicate the study's design with a commonly used term in the title or the abstract	2
		(*b*) Provide in the abstract an informative and balanced summary of what was done and what was found	2
**Introduction**			
Background/rationale	2	Explain the scientific background and rationale for the investigation being reported	4
Objectives	3	State specific objectives, including any prespecified hypotheses	4
**Methods**			
Study design	4	Present key elements of study design early in the paper	6
Setting	5	Describe the setting, locations, and relevant dates, including periods of recruitment, exposure, follow-up, and data collection	6
Participants	6	(*a*) Give the eligibility criteria, and the sources and methods of selection of participants. Describe methods of follow-up	6
		(*b*) For matched studies, give matching criteria and number of exposed and unexposed	n/a
Variables	7	Clearly define all outcomes, exposures, predictors, potential confounders, and effect modifiers. Give diagnostic criteria, if applicable	6,7
Data sources/ measurement	8*	For each variable of interest, give sources of data and details of methods of assessment (measurement). Describe comparability of assessment methods if there is more than one group	6,7
Bias	9	Describe any efforts to address potential sources of bias	7
Study size	10	Explain how the study size was arrived at	6
Quantitative variables	11	Explain how quantitative variables were handled in the analyses. If applicable, describe which groupings were chosen and why	8
Statistical methods	12	(*a*) Describe all statistical methods, including those used to control for confounding	8
		(*b*) Describe any methods used to examine subgroups and interactions	8
		(*c*) Explain how missing data were addressed	8
		(*d*) If applicable, explain how loss to follow-up was addressed	
		(*e*) Describe any sensitivity analyses	8
**Results**			
Participants	13*	(a) Report numbers of individuals at each stage of study—eg numbers potentially eligible, examined for eligibility, confirmed eligible, included in the study, completing follow-up, and analysed	10
		(b) Give reasons for non-participation at each stage	10
		(c) Consider use of a flow diagram	10
Descriptive data	14*	(a) Give characteristics of study participants (eg demographic, clinical, social) and information on exposures and potential confounders	10
		(b) Indicate number of participants with missing data for each variable of interest	10
		(c) Summarise follow-up time (eg, average and total amount)	10
Outcome data	15*	Report numbers of outcome events or summary measures over time	10
Main results	16	(*a*) Give unadjusted estimates and, if applicable, confounder-adjusted estimates and their precision (eg, 95% confidence interval). Make clear	23

For peer review only

		which confounders were adjusted for and why they were included	
		(*b*) Report category boundaries when continuous variables were categorized	n/a
		(*c*) If relevant, consider translating estimates of relative risk into absolute risk for a meaningful time period	n/a
Other analyses	17	Report other analyses done—eg analyses of subgroups and interactions, and sensitivity analyses	11,12
**Discussion**			
Key results	18	Summarise key results with reference to study objectives	13
Limitations	19	Discuss limitations of the study, taking into account sources of potential bias or imprecision. Discuss both direction and magnitude of any potential bias	13
Interpretation	20	Give a cautious overall interpretation of results considering objectives, limitations, multiplicity of analyses, results from similar studies, and other relevant evidence	16
Generalisability	21	Discuss the generalisability (external validity) of the study results	14
**Other information**			
Funding	22	Give the source of funding and the role of the funders for the present study and, if applicable, for the original study on which the present article is based	17

*Give information separately for exposed and unexposed groups.

Note: An Explanation and Elaboration article discusses each checklist item and gives methodological background and published examples of transparent reporting. The STROBE checklist is best used in conjunction with this article (freely available on the Web sites of PLoS Medicine at http://www.plosmedicine.org/, Annals of Internal Medicine at http://www.annals.org/, and Epidemiology at http://www.epidem.com/). Information on the STROBE Initiative is available at http://www.strobe-statement.org.

For peer review only

BMJ Open

Are objective measures of physical capability related to accelerated epigenetic age? Findings from a British birth cohort.

Journal:	*BMJ Open*
Manuscript ID	bmjopen-2017-016708.R1
Article Type:	Research
Date Submitted by the Author:	08-Jun-2017
Complete List of Authors:	Simpkin, Andrew; MRC Integrative Epidemiology Unit, ; University of Bristol<br>Cooper, Rachel; UCL, MRC Unit for Lifelong Health and Ageing at UCL<br>Howe, Laura; University of Bristol, Social Medicine<br>Relton, Caroline; University of Bristol,<br>Davey Smith, George; University of Bristol, Social Medicine<br>Teschendorff, Andrew; UCL<br>Widschwendter, Martin; University College Hospital, 2Department of Women's Cancer<br>Wong, Andrew; UCL, MRC Unit for Lifelong Ageing<br>Kuh, Diana; MRC Unit for Lifelong Health and Ageing at UCL,<br>Hardy, Rebecca; University College London
**Primary Subject Heading**:	Epidemiology
Secondary Subject Heading:	Genetics and genomics
Keywords:	ALSPAC, DNA methylation, epigenetic age, NSHD, physical capability

SCHOLARONE™ Manuscripts

Are objective measures of physical capability related to accelerated epigenetic age? Findings from a British birth cohort.

Andrew J Simpkin[1], Rachel Cooper[2], Laura D Howe[1], Caroline L Relton[1], George Davey Smith[1], Andrew Tescendorff[3,4,5], Martin Widschwendter[3], Andrew Wong[2], Diana Kuh[2], Rebecca Hardy[2]*

1. MRC Integrative Epidemiology Unit, School of Social and Community Medicine, University of Bristol, Bristol BS8 2PS, UK

2. MRC Unit for Lifelong Health and Ageing at UCL, London WC1B 5JU, UK

3. Department of Women's Cancer, University College London, Medical School Building, Room 340, 74 Huntley Street, London WC1E 6AU, UK

4. CAS Key Lab of Computational Biology, CAS-MPG Partner Institute for Computational Biology, Shanghai Institute for Biological Sciences, Chinese Academy of Sciences, Shanghai 200031, China

5. Statistical Cancer Genomics, Paul O'Gorman Building, UCL Cancer Institute, University College London, 72 Huntley Street, London WC1E 6BT, UK

* Corresponding author

Address:

Barley House

Oakfield Road

Clifton

Bristol BS8 2PS

United Kingdom

Email: andrew.simpkin@bristol.ac.uk

Phone: +44 117 33 14026

Fax: +44 117 33 10123

Word count: 3547

Keywords: ALSPAC, DNA methylation, epigenetic age, NSHD, physical capability

For peer review only - http://bmjopen.bmj.com/site/about/guidelines.xhtml

Abstract

Objectives: Our aim was to investigate the association of epigenetic age and physical capability in later life. Having a higher epigenetic than chronological age (known as age acceleration, AA) has been found to be associated with an increased rate of mortality. Similarly, physical capability has been proposed as a marker of ageing due to its consistent associations with mortality.

Setting: The MRC National Survey of Health and Development (NSHD) cohort study.

Participants: We used data from 790 women from the NSHD who had DNA methylation data available.

Design: Epigenetic age was calculated using buccal cell (n=790) and matched blood tissue (n=152) from 790 female NSHD participants. We investigated the association of AA at age 53 with changes in physical capability in women from ages 53 to 60-64. Regression models of change in each measure of physical capability on AA were conducted. Secondary analysis focussed on the relationship between AA and smoking, alcohol, body mass index (BMI) and socioeconomic position.

Outcome measures: Three objective measures of physical capability were used: grip strength, standing balance time and chair rise speed.

Results: Epigenetic age was lower than chronological age (mean 53.4) for both blood (50.3) and buccal cells (42.8). AA from blood was associated with a greater decrease in grip strength from age 53 to 60-64 (0.42kg decrease per year of AA, 95% confidence interval 0.03, 0.82kg; p=0.03, n=152), but no associations were observed with standing balance time or chair rise speed. Current smoking and lower BMI were associated with lower epigenetic age from buccal cells.

Conclusions: We found evidence that AA in blood is associated with a greater decrease in grip strength in British females between 53 and 60-64, but no association with standing balance time or chair rise speed was found.

For peer review only - http://bmjopen.bmj.com/site/about/guidelines.xhtml

Strengths and limitations of this study

- Our study is one of the first to examine epigenetic age from different tissues on the same individuals in relation to objective measures of physical capability, which are key markers of healthy ageing.
- We used serial measures of physical capability on the same individuals over time, allowing for better inferences on changes in physical capability in late midlife, compared with having cross sectional data.
- A limitation of our findings is the lack of generalisability - the subsample of the cohort consisted of females only with repeated measures at ages 53 and 60-64 and was restricted to those with complete information on particular variables of interest (i.e. blood and buccal samples).

view only

For peer review only - http://bmjopen.bmj.com/site/about/guidelines.xhtml

INTRODUCTION

There has been considerable recent interest in epigenetic biomarkers of ageing[1-6], which use an individual's DNA methylation data to estimate their "epigenetic age", a concept that could be considered a form of biological age. The Horvath age estimation method[1] found a correlation of 0.96 between chronological and epigenetic age, with individual estimates of epigenetic age within 3 years of chronological age on average. Epigenetic age has the potential to assess our biological age, but little is known about its relationship with our basic physiology. Moreover, several recent papers have found that the difference between epigenetic and chronological age (known as age acceleration, denoted AA) has biological significance. A positive AA indicates an individual's epigenetic age is ahead of their chronological age, a negative AA (i.e. age deceleration) suggests an individual has younger epigenetic age than chronological age. For example, positive AA has been found to be associated with obesity[7], Down's syndrome[8], HIV[9], menopause[10], and all-cause mortality[11 12].

Lower physical capability, assessed using objective measures such as grip strength, chair rise speed and standing balance time have been found to be associated with all-cause mortality[13]. These findings, established through a systematic review of mainly older populations, were also observed using data on physical capability in midlife from the MRC National Survey of Health and Development (NSHD)[14-16], which has followed 5362 individuals born in the same week of March 1946.

It is pertinent to understand the mechanisms underpinning the association between epigenetic age and mortality, since epigenetics are a potentially modifiable risk factor. A recent study identified a cross-sectional association between epigenetic age acceleration and lower grip strength in an older population using data from the Lothian Birth Cohort 1936[17]. There was no strong evidence for links between epigenetic age acceleration and changes in grip strength, lung function or cognition from age 70 to 76. More recent studies have reported no evidence between epigenetic age and either cognitive[18] or composite measures of biomarker [19] of ageing. In the present article we sought to

For peer review only - http://bmjopen.bmj.com/site/about/guidelines.xhtml

investigate the associations between epigenetic age acceleration at age 53 and objective measures of physical capability at ages 53 and 60-64 in the NSHD. We hypothesised that individuals with positive age acceleration (i.e. with epigenetic age higher than expected based on a linear regression of epigenetic age on chronological age) would have lower average physical capability scores and greater declines than those with lower epigenetic age. We also use data from the NSHD to investigate whether increased epigenetic age is associated with mortality risk factors; smoking[20], higher body mass index (BMI)[21 22] and more disadvantaged socioeconomic position (SEP)[23].

For peer review only

For peer review only - http://bmjopen.bmj.com/site/about/guidelines.xhtml

METHODS

Study participants

DNA was first collected in NSHD participants at age 53[24] in 1999; following QC, the study sample with epigenetic information available consisted of 790 women who had a buccal cell sample taken at age 53 (mean age 53.4, standard deviation 0.16, range 53 to 54)), and who had complete information on epidemiological variables of interest and follow-up. Among these 790 women there were 152 who also had epigenetic information available from blood at age 53. These 152 were originally selected for a case-control study of cancer, and consisted of 75 incident cancer cases after age 53 and 77 controls randomly selected from those with complete data available for the cancer study [16 25 26]. Mortality risk factor data were available at age 53 on smoking status (current, never or ex-smoker), nurse measured height (cm) and weight (kg) (used to calculate BMI (kg/m^2)). Childhood socio-economic position (SEP) was indicated by father's occupational class and SEP in adulthood by own occupational social class at 53? and educational qualifications by age 26. Father's occupational class and own occupational class in adulthood were each defined according to the Registrar General's social classification: unskilled, partly skilled, skilled (manual), skilled (non-manual), intermediate or professional. Education level attained was classified as none, vocational, sub General Certificate of Secondary Education (GCSE), O level, A level, degree or higher.

Study outcomes

The three measures of physical capability were grip strength (kg), standing balance time (seconds) and chair rise time (seconds) measured at age 53 and again at age 60-64 by nurses using standardised protocols[27]. Grip strength was ascertained isometrically using an electronic dynamometer which was calibrated using a back-loading rig and was stable to within 0.5kg. Two values from each hand were recorded at 53, and three in each hand at 60-64, with the maximum of the first four values at each age used for analysis. The standing balance test recorded the times that participants could stand on one leg up to a maximum of 30 seconds first with eyes open and then

For peer review only - http://bmjopen.bmj.com/site/about/guidelines.xhtml

repeated with eyes closed. Balance times with eyes closed were used for analysis and these were log transformed to reduce skewness. Chair rise time was measured using a stopwatch and recorded as the time taken to rise from a seated position to a standing position with a straight back and legs followed by a return to a seated position as fast as possible, repeated 10 times. Chair rise speed was then calculated by dividing the number of rises (i.e. 10) by the time taken in minutes. This was done to make high scores correspond to good performance, as for the other two measures. Nurses recorded whether the participant was unwilling or unable to perform each of the tests along with the reason for this.

Composite capability scores were generated by combining performance on grip strength, balance time and chair rise speed using methods previously described[14]. In brief, each measure was rescaled to a 0 (low) – 1 (high) scale before aggregation into a composite score from 0-3 at ages 53 and 60-64. Standing balance time was rescaled by dividing by 30 seconds (the maximum time allowed); height adjusted grip strength and chair rise time were rescaled by dividing by the 99th percentile. Those unable to carry out a test for health reasons were assigned a score of 0 for that test.

DNA methylation data

DNA methylation was measured using the Infinium HumanMethylation450 BeadChip (Illumina, Inc) in NSHD participants who had biological samples collected in 1999; 638 (buccal cell only) and 152 (buccal cell and blood) [25]. Quality control (QC) and normalisation was performed on each of the 790 buccal samples and then separately on the 152 matched whole blood samples. For each, the minfi package was used to process raw .idat data files[28], using the Illumina definition of beta-values and extracting p-values of detection for each sample. The Illumina methylation beta-value of a given CpG site is found from the intensity of the methylated (M) and unmethylated (U) alleles, as the ratio of fluorescent signals β=Max(M,0)/[Max(M,0)+Max(U,0)+100]. The level of methylation is expressed as a "beta" value (β-value), ranging from 0 (no cytosine methylation) to 1 (complete cytosine methylation). As a further QC step, probes that contained <95% of signals detectable above

For peer review only - http://bmjopen.bmj.com/site/about/guidelines.xhtml

background signal (detection p-value<0.01) were removed from further analysis, and the rest of missing values were imputed using the k-nearest neighbours imputation procedure[29]. To correct for the well-known bias of type-2 probes, we used the subset-quantile within normal array (SWAN) package[30]. To check robustness of this correction procedure, we verified that results were largely unchanged using beta mixture quantile normalistation (BMIQ)[31]. This completed the intra-sample normalization. All participants provided written informed consent. The Central Manchester Ethics Committee approved the use of these samples for epigenetic studies of health.

Epigenetic age

Using the online epigenetic clock calculator (http://labs.genetics.ucla.edu/horvath/dnamage), we obtained DNA methylation estimated age using the Horvath[1] method. The raw DNA methylation β-values were generated from the 152 blood and 790 buccal cell samples. Along with epigenetic age, the online calculator estimates raw age acceleration differences (epigenetic minus chronological age) and age acceleration residuals (the residuals from a linear regression of epigenetic age on chronological age). Our main exposure of interest is the latter age acceleration residual, which we will call age acceleration and denote AA. AA values from blood were corrected for estimated cell type heterogeneity using the Houseman method[32]. The Houseman estimated cell counts were included in the regression of epigenetic age on chronological age to get cell count adjusted AA.

Statistical analysis

Median absolute error was used to investigate the relationship between chronological age and epigenetic age from blood and buccal tissue, with correlation being secondary given the low range of actual age in NSHD. Changes in each physical capability measure were considered the main outcomes, with the differences in grip strength, chair rise speed, balance time and composite score from age 53 to age 60-64 being used for analysis. Using this unconditional change model allows us to directly compare our results with those from the Lothian Birth Cohort 1936[17]. The differences were regressed on AA from blood and buccal tissue separately. We fitted unadjusted regression models

For peer review only - http://bmjopen.bmj.com/site/about/guidelines.xhtml

followed by models adjusted for age, height and BMI and then additionally adjusted for smoking and both childhood and adult SEP. Linear regression was also used to test the association of AA (from both blood and buccal cells) at age 53 with each physical capability measure and the composite score at both ages 53 and 60-64. As a secondary analysis with AA as the outcome, we carried out unadjusted regression analysis of the known mortality risk factors of height, BMI, smoking and SEP (both childhood and adult).

In a sensitivity analysis, we reran the main models with inclusion of those women who were unable to perform each of the three physical capability tests for health reasons (Table 1 includes percentage unable to perform each task). To enable their inclusion, women who were unable to complete a test for health reasons were allocated the minimum value observed at either age.

Replication

Findings were tested for replication using cross sectional data from the mothers of the Avon Longitudinal Study of Parents and Children (ALSPAC)[33 34]. ALSPAC recruited 14541 pregnant women with expected delivery dates between April 1991 and December 1992. Of these initial pregnancies there were 14062 live births and 13988 children who were alive at one year of age. The study website contains details of all the data that are available through a fully searchable data dictionary (http://www.bris.ac.uk/alspac/researchers/data-access/data-dictionary). DNA methylation and epigenetic age were available from 988 ALSPAC mothers at mean age of 46.9 (standard deviation 4.7 years, range 31 to 60) as part of the Accessible Resource for Integrated Epigenetics Studies (ARIES) project[35]. The ARIES study is a subsample of ALSPAC which generated DNA methylation for 1000 families who had biological samples available at each of five time points: umbilical cord blood at birth, peripheral blood at age 7 and 17 in children, and peripheral blood during pregnancy and at 18 years' follow-up for mothers. The 1000 families were randomly selected from those who had full data available. We used the mother's follow-up data to replicate our analysis because they best reflected the available NSHD women. All DNA methylation wet-lab and pre-processing analyses were

For peer review only - http://bmjopen.bmj.com/site/about/guidelines.xhtml

performed at the University of Bristol as part of the ARIES project. Following extraction, DNA was bisulphite converted using the Zymo EZ DNA MethylationTM kit (Zymo, Irvine, CA). Infinium HumanMethylation450 BeadChips were used to measure genome-wide DNA methylation levels at over 485,000 CpG sites. The arrays were scanned using an Illumina iScan, with initial quality review using GenomeStudio. The assay detects methylation of cytosine at CpG islands using two site-specific probes – one to detect the methylated (M) locus and one to detect the unmethylated (U) locus. Single-base extension of the probes incorporates a labelled chain-terminating ddNTP, which is then stained with a fluorescence reagent. The ratio of fluorescent signals from the methylated site versus the unmethylated site determines the level of methylation at the locus. The level of methylation is expressed as a "beta" value (β-value), ranging from 0 (no cytosine methylation) to 1 (complete cytosine methylation). β-values are reported as percentages.

Grip strength, balance time and chair rise speed along with height, BMI, smoking and SEP (adulthood only) were available from these same women. Grip strength was assessed using the Jamar handgrip dynamometer and was recorded to the nearest 1kg using both the right and left hands. Two measures were taken in each hand and the maximum of these values was used. In the chair rise test, the participant was asked to rise from a sitting position to a straight-legged fully standing position five times while being timed. Chair rise speed was then calculated by dividing five by the total time required. This differs from NSHD in having five total stands, though most of the between study variability would be resolved by using chair rise speed rather than total time taken. In the balance time test, the participant stood next to a table and asked to choose a leg and raise it off the floor to ankle height. The participant was timed until they lost their balance and dropped their foot or had to reach out to the table for support. If the participant remained on one leg for longer than 30 seconds they were stopped. The process was repeated with eyes closed, which was used for analysis to mirror the NSHD measure.

For peer review only - http://bmjopen.bmj.com/site/about/guidelines.xhtml

RESULTS

The descriptive statistics are displayed in Table 1. The average epigenetic age was 42.8 (SD 5.71 years) using DNA methylation from buccal tissue and 50.3 (SD 4.34 years) using DNA methylation from blood. These both underestimate the average chronological age of 53.4 years (SD 0.16 years). The median absolute error between chronological and epigenetic age is 10.5 and 4.1 years for buccal and blood respectively. Correlation with chronological age was much lower than previously reported: 0.022 (p=0.79) for blood age and 0.115 (p=0.16) for buccal age, but this correlation is a less appropriate assessment due to the narrow age range (SD of age=0.16 years). Correlation was slightly higher between the two epigenetic ages, with a Pearson correlation coefficient of 0.190 (p=0.02).

Age acceleration (AA), being the residual of a regression of epigenetic age on chronological age, has a mean close to zero by definition. However the variance and range are larger for AA of buccal tissue compared to blood tissue. Average levels of each physical capability measure changed in the expected direction, with a mean decrease in grip strength of 2.6kg (SD 8.5kg), chair rise speed of 6.8 stands/minute (SD 10.1 stands/min) and balance time of 0.27 log-seconds (SD 0.68 log secs) from age 53 to 60-64. This is reflected in an average decrease of 0.12 units in the composite score for physical capability.

Age acceleration and physical capability

Change in physical capability

For a 1-year increase in AA, grip strength decreased by an additional 0.42kg (95% CI 0.03, 0.82kg; p=0.03) from age 53 to 60-64 after adjusting for height, BMI, education and SEP (Table 2). There was no strong evidence for an association between AA and change in chair rise speed (0.06 higher stands per minute per 1-year AA 95% CI -0.40, 0.52 stands per minute; p=0.80) or balance time (0.01 log-seconds lower per 1-year AA, 95% CI -0.04, 0.02 log-seconds; p=0.55). The weak associations of AA from buccal cells and physical capability were in the expected direction.

For peer review only - http://bmjopen.bmj.com/site/about/guidelines.xhtml

Separate analysis of physical capability measured at 53 and 60-64

There were no associations between physical capability at age 53 and epigenetic age acceleration, either from blood or buccal samples (Table S1). Effect sizes were much smaller in buccal AA compared with blood AA for grip strength and particularly for chair rise speed. The associations of AA with grip strength, balance time and the composite score were positive, indicating greater AA was associated with better performance, i.e. the opposite direction to that expected. Similarly, there was little evidence for an association between epigenetic age and any of the physical capability markers or the composite score at age 60-64 (Table S2). Here the effect of AA was in the expected negative direction for grip strength, chair rise speed and balance time, with stronger effects observed from blood AA than buccal AA.

Age acceleration and mortality risk factors

There were positive associations between BMI at age 53 and AA from buccal tissue: 0.085 (95% CI: 0.014, 0.156; p=0.02) years of AA per 1kg/m^2 increase in BMI. The strength of association was lower for blood tissue: 0.044 years of AA per 1kg/m^2 change in BMI (95% CI: -0.065, 0.154 years; p=0.42) (Table 3). There was no association between height and AA. We observed an association between smoking and AA of buccal tissue (p=0.001), but not in blood tissue. Current smokers had the lowest AA on average, with ex- and never-smokers having 1.88 (95% CI 0.85, 2.9) and 1.85 (95% CI 0.76, 3.0) extra years of AA. AA did not vary by childhood or adult SEP.

Sensitivity analysis

We provide associations of AA and change in physical capability when including imputed data for those unable to perform tests in Table S3. Including individuals unable to perform the grip strength test attenuates its association with AA. For a 1-year increase in AA, grip strength decreased by an additional 0.29kg (95% CI -0.74, 0.15kg; p=0.19) from age 53 to 60-64 in a fully adjusted model.

For peer review only - http://bmjopen.bmj.com/site/about/guidelines.xhtml

Including individuals unable to perform tasks did not dramatically affect any of the other blood AA or buccal associations.

Replication

Using data from 988 ALSPAC women with mean age 46.9, we attempted to replicate the cross-sectional findings from NSHD (Tables 4 and 5). The median absolute error and correlation between epigenetic and chronological age was 3.9 years and 0.53 in ALSPAC respectively, where the chronological age was 47.4 (standard deviation 4.5 years, range 34.5 to 60). AA was not related to grip strength, chair rise speed or balance time. The finding that higher BMI was associated with greater AA was replicated in the ALSPAC women (0.129 years per 1kg/m^2 increase in BMI, 95% CI 0.051, 0.207 years, p=0.001) but smoking was not associated with AA (p=0.43), although the direction of effect was the same, with ex-smokers having 0.56 years higher AA and never smokers 0.17 years higher AA compared to current smokers on average. As in NSHD, height and education were not associated with AA.

For peer review only - http://bmjopen.bmj.com/site/about/guidelines.xhtml

For peer review only

DISCUSSION

Age acceleration in blood is associated with a greater decline in grip strength from age 53 to 60-64; for every one-year of AA, women had a 0.4kg greater decrease in grip strength. Neither blood nor buccal epigenetic age acceleration at age 53 was associated with grip strength or other measures of physical capability at either 53 or 60-64. The epigenetic age calculated in our sample was systematically lower than chronological age, particularly for buccal cells (mean difference of 10.7 years for buccal cells).

Our study is one of the first to examine epigenetic age from different tissues on the same individuals in relation to risk factors for mortality. We used serial measures of physical capability on the same individuals over time, allowing for better inferences on changes in physical capability in late midlife, compared with having just cross sectional data. However, one limitation is having just two measures, which are susceptible to regression to the mean. A limitation of our findings is the lack of generalisability - the subsample of the cohort consisted of females only with repeated measures at ages 53 and 64 and was restricted to those with complete information on particular variables of interest (i.e. blood and buccal samples). The 790 women sampled here had marginally lower grip strength (25.7kg vs 26.0kg, p=0.4) and chair rise speed (24.5 stands/min vs 25.5 stands/min, p=0.007) than NSHD women overall at age 60-64[36]. Including those individuals who were unable to perform the grip strength test attenuated the association with blood AA. However, although 18 and 21 grip strength tests could not be performed at 53 and 60-64 respectively (Table 1), just six of these were from individuals with blood DNA methylation available. The attenuation was mainly due to a single individual with low AA and high grip strength at age 53 who was unable to perform the test at age 60-64. Our results should be viewed with consideration for multiple testing. Our primary analysis includes epigenetic age acceleration from two tissues tested against four measures of physical capability, giving a total of eight tests. This diminishes the strength of the evidence provided by this study.

For peer review only - http://bmjopen.bmj.com/site/about/guidelines.xhtml

Our blood results should be compared to another recent study of epigenetic age and physical capability in an older UK birth cohort[17]. Using data from the Lothian Birth Cohort 1936, cross sectional associations at age 70 were found between greater epigenetic age and weaker grip strength as well as with lower lung function and cognitive capability, but not walking speed; however they found no association between baseline epigenetic age and changes in either physical or cognitive capability from age 70 to 76. We have found some evidence that epigenetic age measured at 53 may be associated with a greater decline in grip strength between 53 and 60-64, but no associations were identified with any physical capability measure at 53 or 60-64. In the Lothian Birth Cohort, the reported effect size of AA on grip strength at age 70 was -0.05kg per year of blood AA with a sample size of 1004[17]. In our analysis the effect sizes at 53 and 60-64 were 0.18kg and -0.15kg per year of blood AA respectively with a sample size of 152. The relatively small sample in our analysis of blood AA may mean our study lacks the required power, or it could be that this association only manifests at older ages. It is possible that the association is beginning to emerge in NSHD at 60-64 (an age still younger than the Lothian Birth Cohort baseline) with the suggestion of faster rates of change in those with greater AA. We do not, however, observe any associations between buccal cell AA and physical capability where we have a larger sample size and more comparable statistical power to the Lothian Birth Cohort. Our different results could also be due to sex differences between the two studies; the LBC includes both men and women, whereas we have only looked in females. Several studies have identified higher AA in men than in women[37]. To better understand the epigenetic embodiment of physical capability in later life, one might perform epigenome wide analysis of these measures.

The weak correlations found between epigenetic and chronological ages (0.022 for blood, 0.115 for buccal cells) should be considered with the knowledge that the standard deviation of age is 0.16 years (range 53-54 years). Horvath[1], using data from across 82 studies, compared the standard deviation of age measured in each study with the correlation coefficient found in each study between epigenetic and chronological age. He found a correlation of 0.49 between the SD of age

For peer review only - http://bmjopen.bmj.com/site/about/guidelines.xhtml

and the performance of his epigenetic clock (in terms of correlation). Thus, with such a small age range in our sample, it should be no surprise that we find a diminished correlation. In ALSPAC, by comparison, where the SD of age is larger at 4.5 years, the correlation between epigenetic and chronological age is 0.53. Comparing the median absolute error the difference is much smaller, with 4.1 years in NSHD and 3.9 years in ALSPAC. This suggests that while the correlation metric is not suitable for NSHD, the epigenetic clock itself is valid for blood samples.

While the epigenetic clock was trained on observations from individuals from new-born to 100 years of age, it is likely that age specific clocks could improve on Horvath's clock. However, these benefits are negated by the loss of generalisability. It is also required that the relationship between chronological age and epigenetic age is linear. In our sample, there is no evidence against a linear relationship, and the residuals from this model of epigenetic age on chronological age, i.e. the age accelerations themselves, were normally distributed in this older population.

While the very small range of age at which the epigenetic information was taken in our sample explains the low correlation, it does not explain the bias when using the buccal samples. The systematic difference found here appears to be related to tissue specificity, since epigenetic age from blood was closer to chronological age than buccal epigenetic age. The Horvath age estimator was developed using publicly available data covering 51 tissue types (including buccal cells) such that tissue specificity should not result in such an underestimate of chronological age. Three sets of publicly available buccal cell DNA methylation data[38-40] were among those used in the development of the Horvath epigenetic clock, with a reported correlation of 0.9 between chronological and buccal epigenetic age[1]. However these were from 109 adolescents[38], 30 newborns (i.e. ten pairs of MZ and five pairs of DZ twins between birth and 18 months[40]) and ten individuals who were aged 16, 27, 28, 29, 37, 42, 44, 44, 52, 68[39]. The systematic difference (of 10.7 years) may be explained by the lack of overlap in the age at which information from buccal cells was available in our study and those in the

For peer review only - http://bmjopen.bmj.com/site/about/guidelines.xhtml

training dataset used to derive the epigenetic clock. Our study questions the use of the epigenetic clock for buccal samples in females between 53 and 60-64.

There are a growing number of studies comparing DNA methylation from more than one tissue on the same group of individuals [10 25 41], with one of these using the same NSHD data as the current study[10]. One novel application of the epigenetic clock is to estimate epigenetic ages from different tissues on the same individuals. In the current study we have found evidence that buccal samples are epigenetically younger than blood samples in a UK population. We found a weak correlation between epigenetic age from blood and buccal cells in the same individuals (r=0.19). This low correlation may be due to residual confounding. Since the blood samples come from a case-control study within the birth cohort from which the 790 buccal samples were taken, it may be that the poor correlation between tissues is attributable to selection bias. Further comparisons of epigenetic ages from different tissue types in the same individuals may elucidate our findings.

We found both lower BMI and smoking were related to age deceleration, with the BMI finding replicated in blood methylation age from ALSPAC women at mean age 46.9. Previous research has found this same association between higher BMI and AA in liver tissue[7 37], but ours is the first finding in buccal cells. Our finding that smoking is associated with lower AA is unexpected, since previous research suggests that positive blood AA is associated with higher rates of mortality[11]. This could be due to our use of buccal cells, which are likely more reflective of the effect of smoking on DNA methylation[25]. It is currently unknown whether buccal AA is associated with mortality, nor if the direction is the same as blood AA. Further to this point, the smoking and AA association was not seen in blood samples from ALSPAC or NSHD participants, suggesting this particular result may be spurious.

In conclusion, having a higher epigenetic than chronological age is associated with a greater decline in grip strength in British females between 53 and 60-64, but overall there is little evidence that AA is associated with physical capability change in these women. AA does not appear to be related to

For peer review only - http://bmjopen.bmj.com/site/about/guidelines.xhtml

measures of physical capability in women at ages 53 or 60- 64, while BMI appears to be associated with accelerated epigenetic age in this population.

For peer review only

For peer review only - http://bmjopen.bmj.com/site/about/guidelines.xhtml

FUNDING

This work was supported by a grant from the UK Economic and Social Research Council (ES/M010317/1) and by the UK Medical Research Council (programme codes MC_UU_12019/1, MC_UU_12019/2, MC_UU_12019/4). Research reported in this publication was supported by the National Institute on Ageing of the National Institutes of Health under Award No. R01AG048835. AJS was supported by the UK Medical Research Council (grant MR/L011824/1). LDH was supported by a fellowship from the UK Medical Research Council (MR/M020894/1). The UK Medical Research Council and the Wellcome Trust (Grant ref: 102215/2/13/2) and the University of Bristol provide core support for ALSPAC. Collection of ALSPAC epigenetic and phenotypic data were funded by grants from the Wellcome Trust (WT092830M) and Joint UK Research Councils under the *Lifelong Health and Wellbeing - Phase 3 initiative* (G1001357).

ACKNOWLEDGEMENTS

For use of the ALSPAC data, we are extremely grateful to all the families who took part in this study, the midwives for their help in recruiting them, and the whole ALSPAC team, which includes interviewers, computer and laboratory technicians, clerical workers, research scientists, volunteers, managers, receptionists and nurses. Ethical approval for ALSPAC was obtained from the ALSPAC Ethics and Law Committee and the Local Research Ethics Committees. The content is solely the responsibility of the authors and does not necessarily represent the official views of the National Institutes of Health.

CONTRIBUTORSHIP STATEMENT

This publication is the work of the authors and Andrew Simpkin will serve as guarantors for the contents of this paper. AJS performed designed and ran analysis, drafted the manuscript. RC, RH, DK, AW, AT, MW supervised NSHD analysis. GDS, CLR, LDH supervised ALSPAC analysis. All authors contributed to writing the manuscript.

For peer review only - http://bmjopen.bmj.com/site/about/guidelines.xhtml

DATA SHARING STATEMENT

Data used in this publication are available to bona fide researchers upon request to the NSHD Data Sharing Committee via a standard application procedure. Further details can be found at: http://www.nshd.mrc.ac.uk/data; doi: 10.5522/NSHD/Q101; doi: 10.5522/NSHD/Q102.

COMPETING INTERESTS

None declared

REFERENCES

1. Horvath S. DNA methylation age of human tissues and cell types. *Genome Biol* 2013;14(10):R115.
2. Hannum G, Guinney J, Zhao L, et al. Genome-wide methylation profiles reveal quantitative views of human aging rates. *Mol Cell* 2013;49(2):359-67.
3. Bocklandt S, Lin W, Sehl ME, et al. Epigenetic predictor of age. *PLoS One* 2011;6(6):e14821.
4. Jones MJ, Goodman SJ, Kobor MS. DNA methylation and healthy human aging. *Aging cell* 2015:DOI:10.1111/acel.12349.
5. Weidner CI, Lin Q, Koch CM, et al. Aging of blood can be tracked by DNA methylation changes at just three CpG sites. *Genome Biol* 2014;15(2):R24.
6. Zbieć-Piekarska R, Spólnicka M, Kupiec T, et al. Examination of DNA methylation status of the ELOVL2 marker may be useful for human age prediction in forensic science. *Forensic Science International: Genetics* 2015;14:161-67.
7. Horvath S, Erhart W, Brosch M, et al. Obesity accelerates epigenetic aging of human liver. *Proceedings of the National Academy of Sciences* 2014;111(43):15538-43.
8. Horvath S, Garagnani P, Bacalini MG, et al. Accelerated epigenetic aging in Down syndrome. *Aging cell* 2015;14(3):491-95.
9. Horvath S, Levine AJ. HIV-1 infection accelerates age according to the epigenetic clock. *J Infect Dis* 2015:jiv277.
10. Levine ME, Lu AT, Chen BH, et al. Menopause accelerates biological aging. *Proceedings of the National Academy of Sciences* 2016:201604558.
11. Marioni RE, Shah S, McRae AF, et al. DNA methylation age of blood predicts all-cause mortality in later life. *Genome Biol* 2015;16(1):25.
12. Chen BH, Marioni RE, Colicino E, et al. DNA methylation-based measures of biological age: meta-analysis predicting time to death. *Aging (Albany NY)* 2016;8(9):1844.
13. Cooper R, Kuh D, Hardy R. Objectively measured physical capability levels and mortality: systematic review and meta-analysis. *BMJ* 2010;341:c4467.
14. Cooper R, Strand BH, Hardy R, et al. Physical capability in mid-life and survival over 13 years of follow-up: British birth cohort study. *BMJ: British Medical Journal* 2014;348 doi: 10.1136/bmj.g2219
15. Wadsworth M, Kuh D, Richards M, et al. Cohort profile: the 1946 national birth cohort (MRC National Survey of Health and Development). *Int J Epidemiol* 2006;35(1):49-54.
16. Kuh D, Pierce M, Adams J, et al. Cohort profile: updating the cohort profile for the MRC National Survey of Health and Development: a new clinic-based data collection for ageing research. *Int J Epidemiol* 2011;40(1):e1-e9.

For peer review only - http://bmjopen.bmj.com/site/about/guidelines.xhtml

For peer review only

17. Marioni RE, Shah S, McRae AF, et al. The epigenetic clock is correlated with physical and cognitive fitness in the Lothian Birth Cohort 1936. *Int J Epidemiol* 2015:dyu277.
18. Starnawska A, Tan Q, Lenart A, et al. Blood DNA methylation age is not associated with cognitive functioning in middle-aged monozygotic twins. *Neurobiol Aging* 2017;50:60-63.
19. Belsky DW, Moffitt TE, Cohen AA, et al. Telomere, epigenetic clock, and biomarker-composite quantifications of biological aging: Do they measure the same thing? *bioRxiv* 2016:071373.
20. Doll R, Peto R, Boreham J, et al. Mortality in relation to smoking: 50 years' observations on male British doctors. *BMJ* 2004;328(7455):1519.
21. Lawlor DA, Hart CL, Hole DJ, et al. Reverse causality and confounding and the associations of overweight and obesity with mortality. *Obesity* 2006;14(12):2294-304.
22. Smith GD, Hart C, Upton M, et al. Height and risk of death among men and women: aetiological implications of associations with cardiorespiratory disease and cancer mortality. *J Epidemiol Community Health* 2000;54(2):97-103.
23. Smith GD, Hart C, Blane D, et al. Lifetime socioeconomic position and mortality: prospective observational study. *BMJ* 1997;314(7080):547.
24. Rousseau K, Vinall L, Butterworth S, et al. MUC7 haplotype analysis: results from a longitudinal birth cohort support protective effect of the MUC7* 5 allele on respiratory function. *Ann Hum Genet* 2006;70(4):417-27.
25. Teschendorff AE, Yang Z, Wong A, et al. Correlation of Smoking-Associated DNA Methylation Changes in Buccal Cells With DNA Methylation Changes in Epithelial Cancer. *JAMA Oncol* 2015;1(4):476-85. doi: 10.1001/jamaoncol.2015.1053 [published Online First: 2015/07/17]
26. Anjum S, Fourkala E-O, Zikan M, et al. A BRCA1-mutation associated DNA methylation signature in blood cells predicts sporadic breast cancer incidence and survival. *Genome Med* 2014;6(6):47.
27. Kuh D, Bassey EJ, Butterworth S, et al. Grip strength, postural control, and functional leg power in a representative cohort of British men and women: associations with physical activity, health status, and socioeconomic conditions. *The Journals of Gerontology Series A: Biological Sciences and Medical Sciences* 2005;60(2):224-31.
28. Aryee MJ, Jaffe AE, Corrada-Bravo H, et al. Minfi: a flexible and comprehensive Bioconductor package for the analysis of Infinium DNA methylation microarrays. *Bioinformatics* 2014;30(10):1363-69.
29. Troyanskaya O, Cantor M, Sherlock G, et al. Missing value estimation methods for DNA microarrays. *Bioinformatics* 2001;17(6):520-25.
30. Maksimovic J, Gordon L, Oshlack A. SWAN: Subset-quantile within array normalization for illumina infinium HumanMethylation450 BeadChips. *Genome Biol* 2012;13(6):R44.
31. Teschendorff AE, Marabita F, Lechner M, et al. A beta-mixture quantile normalization method for correcting probe design bias in Illumina Infinium 450 k DNA methylation data. *Bioinformatics* 2013;29(2):189-96.
32. Houseman EA, Accomando WP, Koestler DC, et al. DNA methylation arrays as surrogate measures of cell mixture distribution. *BMC Bioinformatics* 2012;13(1):86.
33. Fraser A, Macdonald-Wallis C, Tilling K, et al. Cohort profile: the Avon Longitudinal Study of Parents and Children: ALSPAC mothers cohort. *Int J Epidemiol* 2013;42(1):97-110.
34. Boyd A, Golding J, Macleod J, et al. Cohort profile: the 'children of the 90s'—the index offspring of the Avon Longitudinal Study of Parents and Children. *Int J Epidemiol* 2012:dys064.
35. Relton CL, Gaunt T, McArdle W, et al. Data Resource Profile: Accessible Resource for Integrated Epigenomic Studies (ARIES). *Int J Epidemiol* 2015:dyv072.
36. Cooper R, Muniz-Terrera G, Kuh D. Associations of behavioural risk factors and health status with changes in physical capability over 10 years of follow-up: the MRC National Survey of Health and Development. *BMJ open* 2016;6(4):e009962.

For peer review only - http://bmjopen.bmj.com/site/about/guidelines.xhtml

37. Simpkin AJ, Hemani G, Suderman M, et al. Prenatal and early life influences on epigenetic age in children: A study of mother-offspring pairs from two cohort studies. *Hum Mol Genet* 2015:ddv456.
38. Essex MJ, Thomas Boyce W, Hertzman C, et al. Epigenetic vestiges of early developmental adversity: childhood stress exposure and DNA methylation in adolescence. *Child Dev* 2013;84(1):58-75.
39. Rakyan VK, Down TA, Maslau S, et al. Human aging-associated DNA hypermethylation occurs preferentially at bivalent chromatin domains. *Genome Res* 2010;20(4):434-39.
40. Martino D, Loke YJ, Gordon L, et al. Longitudinal, genome-scale analysis of DNA methylation in twins from birth to 18 months of age reveals rapid epigenetic change in early life and pair-specific effects of discordance. *Genome Biol* 2013;14(5):R42.
41. Davies MN, Volta M, Pidsley R, et al. Functional annotation of the human brain methylome identifies tissue-specific epigenetic variation across brain and blood. *Genome Biol* 2012;13(6):R43.

For peer review only

For peer review only - http://bmjopen.bmj.com/site/about/guidelines.xhtml

Table 1: Descriptive statistics for continuous variables[1]

Continuous variable	**Age when measured**	**Mean (SD)**	**N**	**N unable (%)**
**Age (years)**	53	53.44 (0.16)	790	-
	64	63.09 (1.09)	623	-
**Epigenetic age from buccal (years)**	53	42.83 (5.71)	790	-
**Age acceleration from buccal (years)**	53	0.00 (5.66)	790	-
**Epigenetic age from blood (years)**	53	50.28 (4.34)	152	-
**Age acceleration from blood (years)**	53	0.00 (4.34)	152	-
**Height (cm)**	53	161.43 (5.61)	790	-
**BMI (kg/m2)**	53	28.13 (6.43)	784	-
	64	29.10 (7.50)	622	-
**Grip strength (kg)**	53	28.18 (8.15)	767	18 (2)
	64	25.71 (7.94)	575	21 (3)
**Grip strength change (kg)**	64	-2.62 (8.53)	560	
**Chair rise speed (stands/minute)**	53	30.98 (9.56)	737	40 (5)
	64	24.54 (7.82)	577	40 (5)
**Chair rise speed change (stands/minute**	64	-6.87 (10.07)	556	
**Balance time (log seconds)**	53	1.77 (0.60)	734	32 (8)
	64	1.52 (0.55)	593	24 (3)
**Balance time change (log seconds)**	64	-0.27 (0.68)	561	-
**Composite score**	53	1.31 (0.33)	750	-
	64	1.19 (0.37)	591	-
**Composite score change**	64	-0.13 (0.36)	558	-
Categorical variable	**Age when measured**	**Category**	**N (%)**	
**Smoking**	53	*Never*	384 (49)	
		Ex	239 (30)	
		Current	167 (21)	
**Childhood SEP**	53	*Professional*	57 (7)	
		Intermediate	160 (21)	
		Skilled (non-manual)	123 (16)	
		Skilled (manual)	245 (31)	
		Partly skilled	148 (19)	
		Unskilled	48 (6)	

[1] BMI = body mass index, GCE = general certificate of education, SEP = socio-economic position.

For peer review only - http://bmjopen.bmj.com/site/about/guidelines.xhtml

**Adult SEP**	53	*Professional*	14 (2)
		Intermediate	261 (33)
		Skilled (non-manual)	286 (36)
		Skilled (manual)	57 (7)
		Partly skilled	119 (15)
		Unskilled	50 (7)
**Education**	53	*None*	275 (35)
		Vocational	44 (5)
		Sub GCE	38 (5)
		O level	201 (26)
		A level	106 (14)
		Burham A2	75 (10)
		Degree	32 (4)
		Postgrad	2 (1)

For peer review only

For peer review only - http://bmjopen.bmj.com/site/about/guidelines.xhtml

Table 2: Association of age acceleration with changes in physical capability from age 53 to 64 in NSHD participants[2]

		**Blood (n=152)**			**Buccal (n=790)**		
**Variable**	Model[3]	Regression Coefficient (difference per year AA)	95% confidence interval	p-value	Regression Coefficient (difference per year AA)	95% confidence interval	p-value
**Grip strength (kg)**	Unadjusted	-0.34	-0.70,0.01	0.06	-0.02	-0.16,0.12	0.73
	Adjusted for age, height, BMI	-0.33	-0.69,0.02	0.06	-0.03	-0.17,0.12	0.73
	Adjusted for height, BMI, smoking, education and SEP	-0.42	-0.82,-0.03	0.03	-0.07	-0.22,0.08	0.35
**Chair rise speed (stands/minute)**	Unadjusted	0.20	-0.23,0.62	0.37	-0.03	-0.17,0.12	0.70
	Adjusted for age, height, BMI	0.19	-0.24,0.62	0.38	-0.04	-0.19,0.10	0.58
	Adjusted for height, BMI, smoking, education and SEP	0.06	-0.40,0.52	0.80	-0.05	-0.20,0.10	0.53
**Balance time, eyes closed (log seconds)**	Unadjusted	-0.01	-0.04,0.02	0.38	-0.001	-0.011,0.010	0.92
	Adjusted for age, height, BMI	-0.01	-0.04,0.02	0.39	-0.002	-0.012,0.009	0.76
	Adjusted for height, BMI, smoking, education and SEP	-0.01	-0.04,0.02	0.55	-0.002	-0.012,0.009	0.73
**Composite score**	Unadjusted	-0.01	-0.028,0.003	0.10	0.001	-0.005,0.007	0.77
	Adjusted for age, height, BMI	-0.01	-0.028,0.003	0.11	0.000	-0.005,0.006	0.87
	Adjusted for height, BMI, smoking, education	-0.01	-0.03,0.01	0.16	-0.001	-0.007,0.005	0.68

[2] AA = age acceleration, BMI = body mass index, SEP = socio-economic position

[3] For each of the four physical capability outcome measures, we ran three models, first unadjusted, then adjusted for height and BMI, then adjusted for height, BMI, smoking, education and both adult and childhood SEP

For peer review only - http://bmjopen.bmj.com/site/about/guidelines.xhtml

	and SEP						

For peer review only

For peer review only - http://bmjopen.bmj.com/site/about/guidelines.xhtml

Table 3: Associations of mortality risk factors with outcome of age acceleration (years) at 53 for NSHD participants[4]

		**Blood (n=152)**			**Buccal (n=790)**		
**Variable**	Level	Regression Coefficient (difference per year AA)	95% confidence interval	p-value	Regression Coefficient (difference per year AA)	95% confidence interval	p-value
**Height (cm)**		-0.017	-0.085,0.051	0.63	-0.011	-0.14,0.11	0.86
**BMI 53 (kg/m^2)**		0.085	0.014,0.16	0.02	0.044	-0.065,0.15	0.42
**Smoking**	Current	Reference		0.001	Reference		0.42
	Ex-smoker	1.88	0.85,2.91		0.83	-0.99,2.66	
	Never	1.86	0.76,2.95		-0.16	-2.17,1.85	
**Childhood SEP**	Professional	Reference		0.80	Reference		0.56
	Intermediate	-0.73	-2.44,0.99		-1.30	-4.01,1.40	
	Skilled (non-manual)	-0.81	-2.59,0.97		-0.48	-3.24,2.27	
	Skilled (manual)	-0.25	-1.89,1.38		-1.24	-3.88,1.39	
	Partly skilled	-0.94	-2.67,0.79		-0.11	-2.90,2.67	
	Unskilled	-0.40	-2.57,1.78		1.39	-2.40,5.17	
**Adult SEP**	Professional	Reference		0.62	Reference		0.35
	Intermediate	0.95	-2.11,4.01		-1.65	-10.27,6.97	
	Skilled (non-manual)	0.95	-2.10,4.00		-1.95	-10.59,6.69	
	Skilled (manual)	1.35	-1.97,4.68		-0.25	-9.13,8.63	
	Partly skilled	0.032	-3.12,3.18		-3.41	-12.31,5.50	
	Unskilled	0.52	-2.85,3.89		0.24	-8.73,9.22	
**Education**	None	Reference		0.78	Reference		0.64
	Vocational	0.16	-1.65,1.98		-1.25	-4.72,2.22	
	Sub GCE	1.20	-0.73,3.14		-1.74	-5.21,1.73	
	O level	0.41	-0.63,1.45		-1.02	-2.90,0.86	
	A level	-0.53	-1.81,0.75		-1.97	-4.29,0.35	
	Burham A2	0.55	-0.91,2.00		-1.43	-3.66,0.81	
	Degree	-0.28	-2.36,1.81		-2.01	-5.73,1.71	
	Postgrad	-0.81	-8.73,7.11		0.84	-0.38,2.06	

[4] AA = age acceleration, BMI = body mass index, GCE = general certificate of education, SEP = socio-economic position

For peer review only - http://bmjopen.bmj.com/site/about/guidelines.xhtml

Table 4: Replication of physical capability and age acceleration in ALSPAC mothers with mean age 46.9[5]

Variable	Model[6]	Regression coefficient (difference per year AA)	95% confidence interval	p-value
**Grip strength (kg)**	Unadjusted	-0.021	-0.12,0.078	0.68
	Adjusted for height, BMI	-0.044	-0.14,0.052	0.37
	Adjusted for height, BMI, SEP	-0.034	-0.13,0.065	0.50
**Chair rise speed (stands/minute)**	Unadjusted	0.0004	-0.0004,0.001	0.31
	Adjusted for height, BMI	0.0005	-0.0003,0.001	0.22
	Adjusted for height, BMI, SEP	0.0004	-0.0004,0.001	0.31
**Balance time, eyes closed (log seconds)**	Unadjusted	0.067	-0.058,0.19	0.30
	Adjusted for height, BMI	0.083	-0.045,0.21	0.20
	Adjusted for height, BMI, SEP	0.088	-0.042,0.22	0.18

Table 5: Replication of mortality risk factors with outcome of age acceleration in ALSPAC mothers with mean age 46.9[7]

Variable	Level	Association with AA (years)	95% confidence interval	p-value
**Height (cm)**		0.0288	-0.034,0.092	0.37
**BMI (kg/m^2)**		0.1293	0.051,0.21	0.001
**Smoking**	Current	Reference		0.43
	Ex	0.5605	-0.88,2.00	
	Never	0.1693	-1.90,2.24	
**Education**	Secondary	Reference		0.28
	Vocational	0.1693	-1.90,2.24	
	O level	0.2586	-1.34,1.86	
	A level	-0.0295	-1.65,1.59	
	Degree	1.2266	-0.47,2.93	

[5] BMI = body mass index, SEP = socio-economic position
[6] For each of the four physical capability measures, we ran three models, first unadjusted, then adjusted for height and BMI, then adjusted for height, BMI, smoking and education
[7] AA = age acceleration, BMI = body mass index

For peer review only - http://bmjopen.bmj.com/site/about/guidelines.xhtml

Table S1: Association of age acceleration with physical capability at age 53 for NSHD participants

		Blood (n=152)			Buccal (n=790)		
**Variable**	Model[1]	Regression Coefficient (difference per year AA)	95% confidence interval	p-value	Regression Coefficient (difference per year AA)	95% confidence interval	p-value
**Grip strength (kg)**	Unadjusted	0.05	-0.25,0.36	0.73	-0.015	-0.12,0.09	0.78
	Adjusted for age, height, BMI	0.07	-0.21,0.36	0.61	-0.004	-0.10,0.10	0.94
	Adjusted for height, BMI, smoking, education and SEP	0.19	-0.14,0.51	0.26	0.010	-0.09,0.11	0.85
**Chair rise speed (stands/minute)**	Unadjusted	-0.29	-0.65,0.07	0.11	0.0001	-0.12,0.12	0.99
	Adjusted for age, height, BMI	-0.25	-0.60,0.09	0.15	0.025	-0.09,0.14	0.68
	Adjusted for height, BMI, smoking, education and SEP	-0.19	-0.57,0.18	0.31	0.002	-0.12,0.12	0.97
**Balance time, eyes closed (log seconds)**	Unadjusted	0.003	-0.02,0.03	0.81	0.003	-0.01,0.01	0.49
	Adjusted for age, height, BMI	0.005	-0.02,0.03	0.69	0.005	-0.003,0.013	0.20
	Adjusted for height, BMI, smoking, education and SEP	0.004	-0.02,0.03	0.71	0.004	-0.003,0.012	0.27
**Composite score**	Unadjusted	0.002	-0.01,0.01	0.77	0.001	-0.003,0.006	0.60
	Adjusted for age, height, BMI	0.003	-0.01,0.02	0.63	0.003	-0.002,0.007	0.22
	Adjusted for height, BMI, smoking, education and SEP	0.01	-0.01,0.02	0.39	0.002	-0.003,0.007	0.38

[1] For each of the four physical capability outcome measures, we ran three models, first unadjusted, then adjusted for height and BMI, then adjusted for height, BMI, smoking, education and both adult and childhood SEP

For peer review only

Table S2: Association of age acceleration at 53 with physical capability at age 60-64 for NSHD participants

		**Blood (n=152)**			**Buccal (n=790)**		
**Variable**	Model[2]	Regression Coefficient (difference per year AA)	95% confidence interval	p-value	Regression Coefficient (difference per year AA)	95% confidence interval	p-value
**Grip strength (kg)**	Unadjusted	-0.18	-0.51,0.15	0.28	-0.08	-0.19,0.04	0.18
	Adjusted for age, height, BMI	-0.18	-0.50,0.14	0.26	-0.06	-0.17,0.05	0.27
	Adjusted for height, BMI, smoking, education and SEP	-0.18	-0.54,0.17	0.30	-0.09	-0.21,0.02	0.12
**Chair rise speed (stands/minute)**	Unadjusted	-0.07	-0.40,0.26	0.67	-0.02	-0.13,0.09	0.75
	Adjusted for age, height, BMI	-0.07	-0.39,0.25	0.66	-0.01	-0.12,0.09	0.82
	Adjusted for height, BMI, smoking, education and SEP	-0.12	-0.45,0.22	0.49	-0.04	-0.15,0.07	0.45
**Balance time, eyes closed (log seconds)**	Unadjusted	-0.009	-0.03,0.01	0.42	-0.0002	-0.01,0.01	0.97
	Adjusted for age, height, BMI	-0.009	-0.03,0.01	0.41	0.001	-0.01,0.01	0.85
	Adjusted for height, BMI, smoking, education and SEP	-0.007	-0.03,0.02	0.53	-0.0005	-0.01,0.01	0.90
**Composite score**	Unadjusted	0.001	-0.004,0.007	0.67	0.001	-0.003,0.006	0.60
	Adjusted for age, height, BMI	0.002	-0.003,0.007	0.54	0.003	-0.002,0.007	0.22
	Adjusted for height, BMI, smoking, education and SEP	0.003	-0.002,0.009	0.25	0.002	-0.003,0.007	0.38

[2] For each of the four physical capability outcome measures, we ran three models, first unadjusted, then adjusted for height and BMI, then adjusted for height, BMI, smoking, education and both adult and childhood SEP

For peer review only

Table S3: Association of age acceleration with changes in physical capability from age 53 to 64 in NSHD participants, including those unable to perform tests

		**Blood (n=152)**			**Buccal (n=790)**		
**Variable**	Model[3]	Regression Coefficient (difference per year AA)	95% confidence interval	p-value	Regression Coefficient (difference per year AA)	95% confidence interval	p-value
**Grip strength (kg)**	Unadjusted	-0.30	-0.69,0.10	0.14	0.02	-0.14,0.17	0.83
	Adjusted for age, height, BMI	-0.28	-0.67,0.11	0.16	0.02	-0.13,0.17	0.80
	Adjusted for height, BMI, smoking, education and SEP	-0.29	-0.73,0.15	0.19	-0.04	-0.20,0.13	0.67
**Chair rise speed (stands/minute)**	Unadjusted	0.21	-0.23,0.64	0.35	-0.01	-0.16,0.14	0.87
	Adjusted for age, height, BMI	0.20	-0.23,0.63	0.36	-0.03	-0.18,0.12	0.74
	Adjusted for height, BMI, smoking, education and SEP	0.07	-0.40,0.54	0.77	-0.04	-0.20,0.12	0.62
**Balance time, eyes closed (log seconds)**	Unadjusted	-0.02	-0.05,0.01	0.21	-0.002	-0.01,0.01	0.77
	Adjusted for age, height, BMI	-0.02	-0.05,0.01	0.21	-0.003	-0.01,0.01	0.65
	Adjusted for height, BMI, smoking, education and SEP	-0.01	-0.05,0.02	0.44	-0.004	-0.02,0.01	0.48

[3] For each of the four physical capability outcome measures, we ran three models, first unadjusted, then adjusted for height and BMI, then adjusted for height, BMI, smoking, education and both adult and childhood SEP

STROBE Statement—Checklist of items that should be included in reports of ***cohort studies***

	Item No	Recommendation	Page No
**Title and abstract**	1	(*a*) Indicate the study's design with a commonly used term in the title or the abstract	2
		(*b*) Provide in the abstract an informative and balanced summary of what was done and what was found	2
**Introduction**			
Background/rationale	2	Explain the scientific background and rationale for the investigation being reported	4
Objectives	3	State specific objectives, including any prespecified hypotheses	4
**Methods**			
Study design	4	Present key elements of study design early in the paper	6
Setting	5	Describe the setting, locations, and relevant dates, including periods of recruitment, exposure, follow-up, and data collection	6
Participants	6	(*a*) Give the eligibility criteria, and the sources and methods of selection of participants. Describe methods of follow-up	6
		(*b*) For matched studies, give matching criteria and number of exposed and unexposed	n/a
Variables	7	Clearly define all outcomes, exposures, predictors, potential confounders, and effect modifiers. Give diagnostic criteria, if applicable	6,7
Data sources/ measurement	8*	For each variable of interest, give sources of data and details of methods of assessment (measurement). Describe comparability of assessment methods if there is more than one group	6,7
Bias	9	Describe any efforts to address potential sources of bias	7
Study size	10	Explain how the study size was arrived at	6
Quantitative variables	11	Explain how quantitative variables were handled in the analyses. If applicable, describe which groupings were chosen and why	8
Statistical methods	12	(*a*) Describe all statistical methods, including those used to control for confounding	8
		(*b*) Describe any methods used to examine subgroups and interactions	8
		(*c*) Explain how missing data were addressed	8
		(*d*) If applicable, explain how loss to follow-up was addressed	
		(*e*) Describe any sensitivity analyses	8
**Results**			
Participants	13*	(a) Report numbers of individuals at each stage of study—eg numbers potentially eligible, examined for eligibility, confirmed eligible, included in the study, completing follow-up, and analysed	10
		(b) Give reasons for non-participation at each stage	10
		(c) Consider use of a flow diagram	10
Descriptive data	14*	(a) Give characteristics of study participants (eg demographic, clinical, social) and information on exposures and potential confounders	10
		(b) Indicate number of participants with missing data for each variable of interest	10
		(c) Summarise follow-up time (eg, average and total amount)	10
Outcome data	15*	Report numbers of outcome events or summary measures over time	10
Main results	16	(*a*) Give unadjusted estimates and, if applicable, confounder-adjusted estimates and their precision (eg, 95% confidence interval). Make clear	23

For peer review only

		which confounders were adjusted for and why they were included	
		(*b*) Report category boundaries when continuous variables were categorized	n/a
		(*c*) If relevant, consider translating estimates of relative risk into absolute risk for a meaningful time period	n/a
Other analyses	17	Report other analyses done—eg analyses of subgroups and interactions, and sensitivity analyses	11,12
**Discussion**			
Key results	18	Summarise key results with reference to study objectives	13
Limitations	19	Discuss limitations of the study, taking into account sources of potential bias or imprecision. Discuss both direction and magnitude of any potential bias	13
Interpretation	20	Give a cautious overall interpretation of results considering objectives, limitations, multiplicity of analyses, results from similar studies, and other relevant evidence	16
Generalisability	21	Discuss the generalisability (external validity) of the study results	14
**Other information**			
Funding	22	Give the source of funding and the role of the funders for the present study and, if applicable, for the original study on which the present article is based	17

*Give information separately for exposed and unexposed groups.

Note: An Explanation and Elaboration article discusses each checklist item and gives methodological background and published examples of transparent reporting. The STROBE checklist is best used in conjunction with this article (freely available on the Web sites of PLoS Medicine at http://www.plosmedicine.org/, Annals of Internal Medicine at http://www.annals.org/, and Epidemiology at http://www.epidem.com/). Information on the STROBE Initiative is available at http://www.strobe-statement.org.

For peer review only

For peer review only - http://bmjopen.bmj.com/site/about/guidelines.xhtml
